# mRNA vaccines: immunogenicity and quality characteristics

**DOI:** 10.1186/s12951-025-03800-5

**Published:** 2025-11-27

**Authors:** Yaru Quan, Huijie Yang, Wei Li, Linxian Li

**Affiliations:** 1 Division of Respiratory Virus Vaccines, National Institutes for Food and Drug Control, NHC Key Laboratory of Research on Quality and Standardization of Biotech Products, NMPA Key Laboratory for Quality Research and Evaluation of Biological Products, Beijing, 102629 China; 2 Walvax Biotechnology Co., Ltd, 395 Kexin Road, High-Tech Zone, Kunming City, Yunnan Province China; 3 Department of Surgery, The Chinese University of Hong Kong, Shatin, Hong Kong; 4 Ming Wai Lau Centre for Reparative Medicine, Karolinska Institutet, Stockholm, 17177 Sweden; 5 Department of Neuroscience, Karolinska Institutet, Stockholm, 17177 Sweden; 6 Center for Neuromusculoskeletal Restorative Medicine, Hong Kong Science Park, New Territories, Shatin, Hong Kong

**Keywords:** mRNA vaccine, Critical quality attributes, Vaccine efficacy, Safety, Vaccine immunogenicity

## Abstract

mRNA vaccine technology platform has evolved rapidly in recent years, which has emerged as an important direction in vaccine development. Establishing a quality control platform based on its Critical Quality Attributes (CQAs) is fundamental to ensuring vaccine efficacy and safety. This paper systematically explores the key quality attributes of mRNA vaccines and their impact on vaccine immunogenicity. Additionally, it introduces the technical principles, development status, key quality control items and their significance, as well as immunogenicity indicators of mRNA vaccines, and discusses the critical importance of studying the correlation between CQAs and immunogenicity.

**Graphical abstract:**

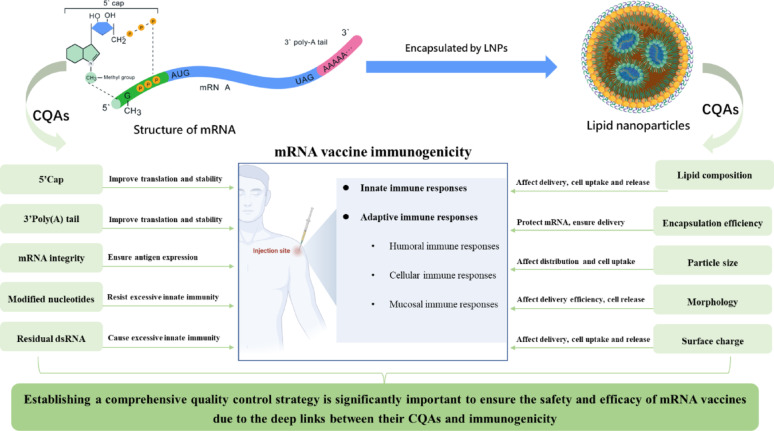

## Introduction

mRNA is a natural biological molecule utilize the host cell’s protein translation machinery to produce target antigens or other functional proteins, thereby providing preventive or therapeutic effects. The successful translation of mRNA vaccine research into clinical applications has depended on systematic breakthroughs in three key areas: delivery vector innovation, sequence modification technologies, and GMP production systems. For example, the incorporation of the modified nucleotide 1-methylpseudouridine in Moderna and BioNTech’s mRNA vaccines significantly reduced the innate immunogenicity of in vitro-synthesized mRNA while enhancing translation efficiency, ensuring optimal intracellular function of mRNA molecules [1, 2]. Furthermore, advances in cationic lipid development and Lipid Nanoparticle (LNP) technology have enabled efficient encapsulation of mRNA molecules and stable in vivo delivery. In optimizing mRNA molecules, co-transcriptional capping technology—particularly the Cap1 structure synthesized using trinucleotide cap analogs—has achieved efficient capping, further improving mRNA translation initiation efficiency [3]. These technological advances have facilitated the successful market launch of multiple mRNA vaccines, including BNT162b2 (BioNTech-Pfizer) and mRNA-1273 (Moderna) for Severe Acute Respiratory Syndrome Coronavirus 2 (SARS-CoV-2), as well as mRNA-1345 (Moderna) for Respiratory Syncytial Virus (RSV).

**Fig. 1:**
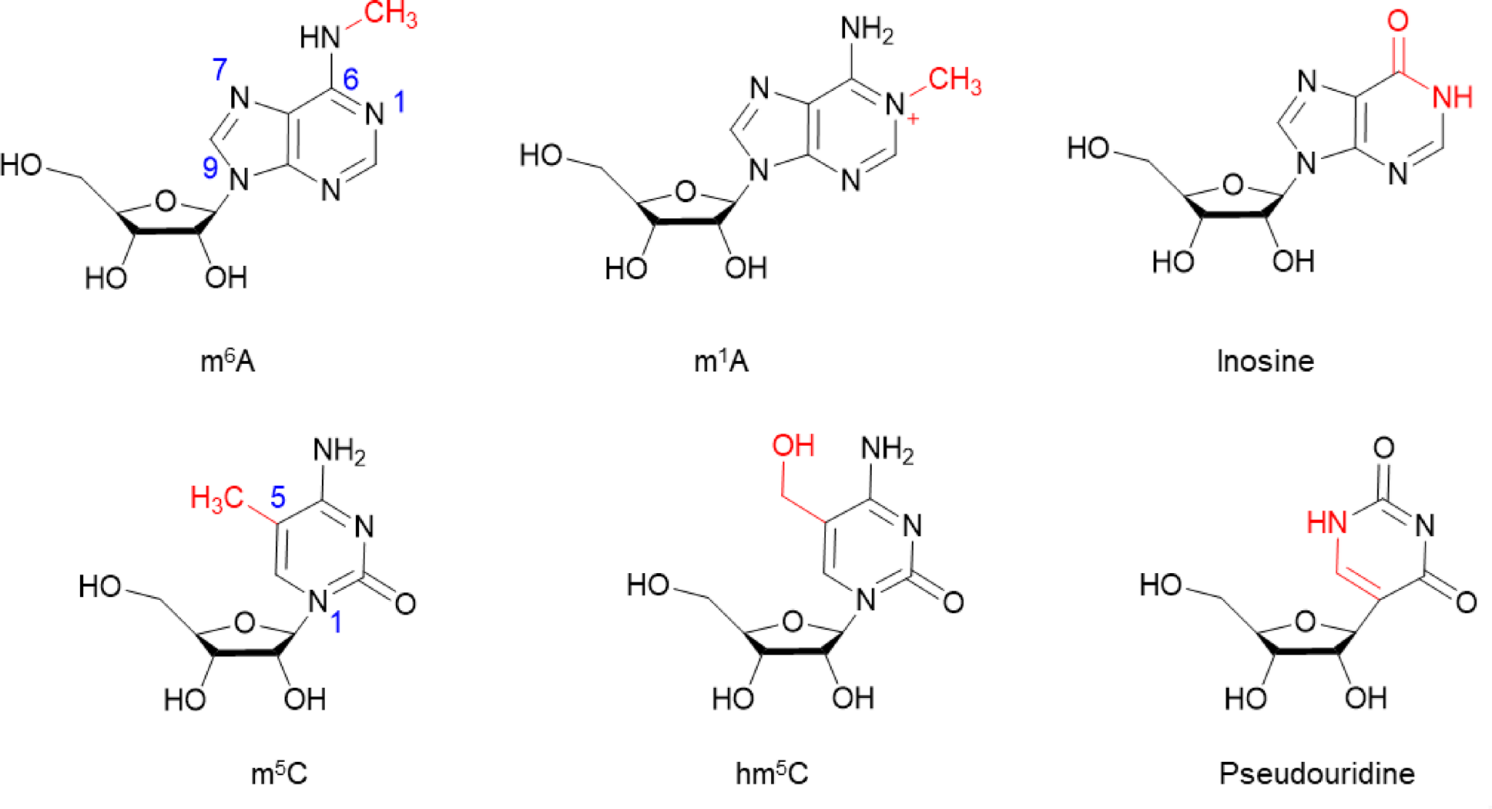
Chemical structure of modified nucleitides in mRNA vaccines

Additionally, the saRNA vaccine ARCT-154**—**jointly developed by Arcturus Therapeutics and CSL**—**for SARS-CoV-2 enhances vaccine efficacy by extending intracellular half-life and has received marketing approval in Japan and the EU. Novel formulation technologies, such as lyophilization, have significantly improved the long-term stability of mRNA vaccines at 4 °C, effectively addressing bottlenecks in cold-chain transportation and storage. Furthermore, new delivery systems have achieved tissue- or organ-specific targeting through optimization of the physicochemical properties of mRNA formulations [4]. **(**Fig. 1**)**

As cutting-edge medical products, research into and effective control of mRNA vaccine quality attributes are directly linked to vaccine safety, efficacy, and quality consistency. According to *the Technical Guidelines for Pharmaceutical Research of mRNA Vaccines for COVID-19 Prevention (Trial)* and relevant United States Pharmacopeia (USP) guidelines, Critical Quality Attributes (CQAs) of mRNA vaccines primarily encompass three categories: mRNA molecular characteristics (including identification, content, integrity, and purity), delivery system properties (such as lipid composition, particle size, encapsulation efficiency, and surface charge), and other general parameters for injectable (including appearance, osmolality, sterility, and pH value). Among these CQAs, mRNA integrity and chemical modifications directly impact antigen expression efficiency and immunogenicity. The physicochemical properties of delivery systems such as LNPs—including particle size and distribution, zeta potential, and lipid ratios—determine in vivo delivery efficiency and targeting specificity. Encapsulation efficiency and other general injectable parameters serve as indispensable monitoring indicators to ensure safe, effective, and quality-controlled vaccine production and administration. Strict control of these CQAs not only influences vaccine immunogenicity (such as total antibody/neutralizing antibody titers and cellular immune responses) but also relates to clinical safety (including local inflammatory reactions and systemic toxicity). Therefore, establishing a multi-dimensional and highly sensitive quality evaluation system serves as the core technical foundation for the industrial application of mRNA vaccines and provides key assurance for ensuring the stability and reliability of their clinical performance.

Vaccine quality directly impacts antigen presentation efficiency, the intensity of innate immune activation, and the level and polarization direction of adaptive immune activation, thereby determining vaccine protective efficacy and safety. mRNA vaccines activate the innate immune system through both delivery systems (such as LNPs) and mRNA molecules, primarily relying on Pattern Recognition Receptors (PRRs) to induce cytokine release, promote Dendritic Cell (DC) maturation and antigen presentation, and subsequently activate adaptive immune responses [5]. Therefore, innate immune assessment should include analysis of cytokines, chemokines, and key innate immune cells (including macrophages, NK cells, and DCs). In terms of adaptive immunity, mRNA vaccines can induce robust humoral and cellular immune responses. These vaccines promote B cell Somatic Hypermutation (SHM) and affinity maturation through germinal center (GC) reactions, generating high-affinity, broadly neutralizing antibodies and long-term memory B cells. They also activate CD4⁺ and CD8⁺ T cells, with cells such as Follicular Helper T cells (Tfh) and Helper T cells (Th) coordinating humoral and cellular immunity to establish long-term immune memory [6, 7].

Consequently, adaptive immunity assessment should encompass both humoral and cellular immunity assays. Specific indicators for humoral immunity include antibody titers, antibody affinity, antibody neutralizing activity and breadth, and temporal kinetic characteristics. Additionally, attention should be given to germinal center formation, including evaluation of Germinal Center B cells (GCB), antigen-specific plasma cells, and memory B cells. For T cell response detection, assessments should include phenotyping of activated T cells (Th1/2/17, etc.), T cell proliferation capacity, and CD8⁺ T cell cytotoxic function. For vaccines administered via mucosal routes or designed for mucosal targeting, detection of secretory IgA and Tissue Resident Memory T cells (TRM) is also required.

## Critical quality attributes (CQAs) of mRNA vaccines

The Critical Quality Attributes (CQAs) of mRNA vaccines are fundamental elements ensuring their safety, efficacy, and successful industrial application. In recent years, global health authorities and regulatory organizations have issued multiple guidelines to standardize and guide critical aspects of mRNA vaccine production, quality control, and clinical/non-clinical evaluation. For example, the World Health Organization (WHO) released the technical document *Regulatory Considerations for the Quality*,* Safety and Efficacy Evaluation of mRNA Vaccines for Infectious Disease Prevention* in December 2020; China’s Center for Drug Evaluation (CDE) published the *Technical Guidelines for COVID-19 Preventive Vaccine Development (Provisional)* in August 2020; the United States Pharmacopeia (USP) updated multiple versions of the *Draft Guidance on Quality Analytical Methods for mRNA Vaccines and Therapeutic Products* between 2022 and 2024; and the European Pharmacopoeia established a series of general rules for mRNA vaccines in April 2024. For mRNA vaccine CQAs, particular attention must be given to the research and quality control of key attributes in the active mRNA bulk solution and LNPs, as well as the characterization and control of CQAs in the final vaccine formulation. The CQAs of mRNA bulk solutions encompass function-related sequences or structural features and purity-related impurities of various types. The CQAs of LNPs encompass lipid composition and physicochemical properties of LNPs. The following section provides a detailed discussion of the significance and common research methods for CQA assessment of mRNA vaccine.

### Structural integrity of mRNA molecules

mRNA integrity is a critical quality attribute that ensures the efficacy, safety, and stability of mRNA vaccines. The integrity of mRNA is essential for its translational activity: intact mRNA enables efficient ribosomal recognition and translation into antigenic proteins [8], while also achieving sustained antigen expression through prolonged half-life, which is crucial for dendritic cells to present antigens via MHC molecules and activate specific T-cell responses [9, 10]. Conversely, fragmented mRNA not only causes translation interruption and reduces antigen production [11], but its degradation products can also be misrecognized by pattern recognition receptors (PRRs) as pathogen signals, triggering premature antiviral responses that hinder translation [12]. Studies demonstrate that incomplete mRNA fragments cannot translate proteins in both cellular and cell-free systems [13]. Additionally, when the 5’ cap structure or 3’ poly(A) tail of mRNA is damaged, ribosome binding efficiency decreases by over 90% [11]. In mouse models, full-length mRNA (>4000 nt) induced antibody titers 5–7 times higher than fragmented mRNA (< 2000 nt) [11], This phenomenon is linked to antigen-presenting cell (APC) function: intact mRNA continuously expresses antigens in dendritic cells for 72 h, while fragmented mRNA lasts only 12–24 h [14].

The integrity of mRNA has a “double-edged sword” effect on innate immunity. The self-adjuvant properties of intact mRNA depend on its interaction with pattern recognition receptors (e.g., TLR3/7/8). However, excessively degraded short RNA fragments may activate the RIG-I/MDA5 pathway, leading to excessive IFN-β secretion and subsequent inhibition of antigen protein translation [15]. Experimental data demonstrate this relationship: when mRNA integrity exceeds 95%, IFN-β levels are positively correlated with neutralizing antibody titers (*r* = 0.72); however, when integrity falls below 80%, this correlation becomes negative (*r*=−0.63) [9].

Currently, two approaches are commonly used to improve mRNA integrity. First, optimizing mRNA sequences can enhance integrity and vaccine stability, reduce excessive immune recognition of exogenous fragments, and balance immunogenicity and safety [16], thereby improving vaccine efficacy and durability. Second, optimizing the LNP system can help maintain mRNA integrity. pH-sensitive lipids (e.g., YSK12-C4) trigger conformational changes in the acidic environment of lysosomes, increasing mRNA release efficiency from 15% to 82% while reducing nuclease exposure time [17]. These systems maintained 92% integrity after 28 days in a 40 °C acceleration test, far exceeding the 65% achieved by traditional LNPs [18].

**Fig. 2:**
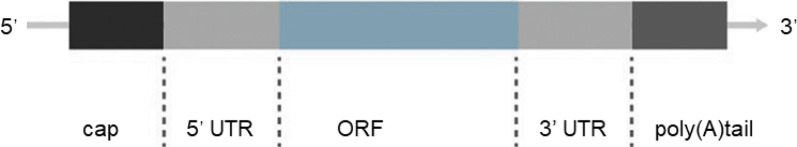
A schematic structure of mRNA vaccine

As shown in Fig. 2, the structural integrity of mRNA molecules comprises five essential components: the 5’ cap, 5’ untranslated region (UTR), coding sequence (CDS), 3’ UTR, and poly(A) tail. Each component plays a critical role in mRNA function, stability, and translation efficiency [19].

#### 5’ cap structure

**Fig. 3:**
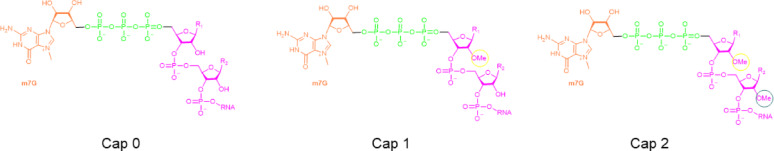
Chemical structure of Cap 0, Cap 1 and Cap2

The 5’ cap structure of mRNA is a specialized modification added to the 5’ end of eukaryotic mRNA, consisting of an N7-methylguanosine (m7G) cap linked to the first transcribed nucleotide. During mRNA formation, the first nucleotide of all mRNAs undergoes 2’-O-methylation (Nm) by cap methyltransferase 1 (CMTR1), forming a Cap1-modified mRNA terminus (m7G-ppp-Nm) (Fig. 3). When mRNA is transported to the cytoplasm, a portion of the Cap1 mRNA undergoes additional 2’-O-methylation at the ribose of the second nucleotide by cap methyltransferase 2 (CMTR2), forming a Cap2-modified 5’ end (m7G-ppp-Nm-Nm) [20].

As early as 1995, Song et al. demonstrated that uncapped Hsp70 mRNA showed approximately 70% lower translation efficiency in an in vitro translation system when studying the cap structure-dependent translation efficiency of Drosophila heat shock protein (HSP70) mRNA [21]. The 5’ cap structure of mRNA plays a vital role in cellular processes, not only initiating translation but also protecting mRNA from nuclease degradation. The cap structure participates in pre-mRNA processing, including splicing, to ensure correct gene expression [22]. It also serves as a recognition signal for mRNA nuclear export. An immunoprecipitation experiment demonstrated that nuclear RNA export factor (REF) interacts with cap-binding protein CBP20, and co-injection of CBP20 and REF enhanced β-globin mRNA export from HeLa cell nuclei [23]. Only capped mRNA can be efficiently exported from the nucleus to participate in subsequent translation [24]. The 5’ cap binds to translation initiation factor eIF4E, recruiting other initiation factors to form a translation initiation complex that triggers mRNA translation [25]. Additionally, the cap structure significantly enhances mRNA stability by preventing nuclease degradation. Under specific conditions (e.g., deadenylation or the presence of nonsense mRNA sequences), mRNA undergoes decapping mediated by the DCP1/2 decapping enzyme complex, followed by rapid degradation via 5’−3’ exonuclease Xrn1, which is directly coupled by EDC4 protein [26]. Thus, the stability of the 5’ cap is critical for regulating mRNA lifespan.

Beyond translation and stability regulation, the 5’ cap structure also plays a key role in reducing innate immune stimulation. Compared with Cap0, the eukaryotic Cap1 structure effectively avoids innate immune responses. Studies demonstrate that the Cap1 structure protects RNA from decapping and degradation by DXO protein, preventing recognition by the innate immune system as non-self RNA, thereby reducing immune activation and improving translation efficiency [27, 28]. Cap1 also inhibits RIG-I recognition of 5’-ppp, reducing IFN secretion [29] and preventing activation of melanoma differentiation-associated protein 5 (MDA5) sensing of long double-stranded RNA (dsRNA) [30]. Abbas et al. demonstrated that only transcripts with an m7G-adjacent nucleotide bearing 2’-O-methylation are completely resistant to interferon-induced tetratricopeptide repeat protein family IFIT1 recognition in vitro [31], and IFIT1 recognition is crucial for distinguishing “self” from “non-self” RNA [32]. Further research revealed that 2’-O-methylation of the first transcribed nucleotide may be insufficient to fully protect mRNA from being recognized as “non-self” by cellular immune defenses [33], suggesting that the presence of additional 2’-O-methylation at the second nucleotide may be a key factor. Transcriptome data also demonstrate that a significant increase in Cap1 abundance leads to RIG-I (retinoic acid-inducible gene I) activation, while Cap1-to-Cap2 methylation significantly reduces RNA binding and RIG-I activation. The slow methylation rate of Cap2 allows its accumulation on mRNA while ensuring low levels on viral RNA in infected cells, revealing that Cap1 has immunostimulatory effects, whereas Cap2 reduces innate immune activation [20].

**Fig. 4:**
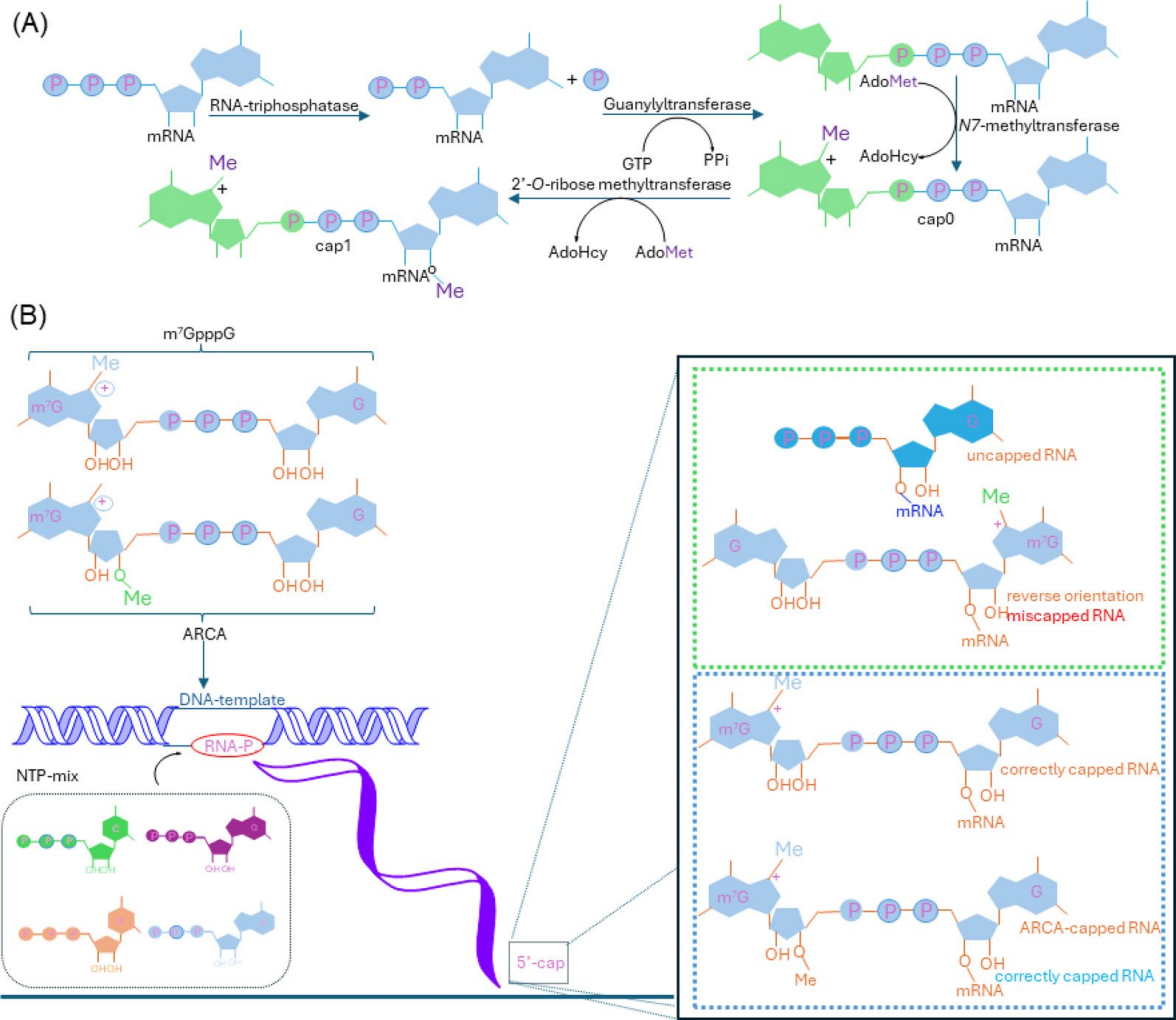
(**A**) Schematic representation of enzymatic 5′-cap formation in eukaryotic mRNA; (**B**) Schematic representation of co-transcriptional capping with different cap analogues

*In vitro transcription (IVT) of mRNA employs two main capping methods: post-transcriptional enzymatic capping and cap analog capping. Enzymatic capping typically uses a vaccinia virus capping enzyme complex to generate a Cap0 structure via a three-step reaction (dephosphorylation*,* guanylylation*,* and N7-methylation)*,* followed by Cap1 formation via 2’-O-methyltransferase* [34] **(**Fig. 4**)**. This method offers high reaction specificity and near-native cap structures but is cumbersome, with capping efficiency highly dependent on the activity of both capping enzyme and 2’-O-methyltransferase. It may also generate multiple intermediate impurities, increasing purification difficulty [35]. In contrast, cap analog capping is more commonly used and has evolved through three technological generations. The first-generation dinucleotide cap analog (m7GpppG) was limited by uncontrolled capping direction, producing inactive reverse caps, with capping efficiency highly dependent on the ratio of cap analog to GTP. The second-generation anti-reverse cap analog (ARCA) solved the reverse cap problem by introducing a methyl group or deoxy group at the 3’ position of the m7G nucleotide but could only generate Cap0, requiring additional 2’-O-methyltransferase treatment for Cap1 formation [36]. The third-generation trinucleotide cap analog (m7GpppAmG, e.g., CleanCap^®^ AG) enables co-transcriptional synthesis of Cap1-structured mRNA without Cap0 intermediates, achieving capping efficiency exceeding 94%. However, further evaluation is needed to determine whether CleanCap^®^ manufacturing processes introduce Cap0 impurities, which would affect final product purity and functionality [3].

#### Poly(A) tail length

The poly(A) tail is a long chain of adenosine residues located in the 3′ untranslated region (UTR) of eukaryotic mRNA, typically consisting of 50–250 adenine (A) nucleotides [37]. It plays a critical role in mRNA stability, translational efficiency, and protein expression [38]. The poly(A) tail protects mRNA from exonuclease degradation by binding to poly(A)-binding proteins (PABP), thereby enhancing mRNA stability [39]. Simultaneously, the poly(A) tail promotes mRNA translation through a sophisticated mechanism: PABPC bound to the poly(A) tail interacts with the translation initiation factor eIF4G, causing the mRNA to form a “closed-loop” structure. This structure facilitates recruitment of the 40S translation initiation complex to the mRNA and works synergistically with the 5’ cap structure, making the presence of the poly(A) tail essential for initiating translation [40]. In contrast, mRNA lacking a poly(A) tail is rapidly degraded, cannot form the initiation complex, and exhibits significantly reduced translational efficiency [41]. Therefore, the poly(A) tail is critical for the mRNA’s life cycle and function, and is considered a critical quality attribute for therapeutic mRNA applications.

Optimizing the length and sequence of the poly(A) tail can improve mRNA stability and translational efficiency, thereby enhancing therapeutic efficacy. Studies have shown that there is a correlation between poly(A) tail length and mRNA translational efficiency, though it is not a simple linear relationship. Biziaev N et al. constructed a luciferase-encoding vector with varying poly(A) tail lengths and conducted experiments in HEK293F cell lysates. The results showed that increasing the poly(A) tail length of capped mRNA to 10 nt led to a 50% increase in translation rate compared to mRNA lacking a poly(A) tail. When increased to 50 nt, translation efficiency remained unchanged; at 75 nt, mRNA displayed significantly enhanced translation efficiency, and at 100 nt, translation efficiency was comparable to the 10–50 nt range. The translation efficiency of uncapped mRNA was only one-tenth that of capped mRNA, but it also increased linearly as the poly(A) tail was extended to 100 nt [40].

Similarly, Mockey et al. constructed luciferase mRNAs with poly(A) tails of 100 nt and 64 nt and measured their luciferase expression levels in mouse dendritic cells (JAWS II). They found that mRNA with a 100 nt tail achieved protein expression efficiency 700 times higher than that of the 64 nt tail [42]. However, some studies have shown that mRNAs with short poly(A) tails (< 20 nt) can also be translated efficiently under specific conditions. Peng J et al. compared the translation of short poly(A) tail mRNAs containing a poly(A)-limiting element (PLE) in cells and the in vitro translation of mRNAs with varying poly(A) tail lengths. In transfected cells, PLE-containing mRNA with fewer than 20 nucleotides in its poly(A) tail was translated similarly to mRNA with a long poly(A) tail and exhibited comparable polysome binding capability. These data suggest that PLE functionally substitutes for bound PABP to stimulate the translation of short poly(A) tail mRNA [43]. The optimal poly(A) tail length may vary depending on the specific mRNA sequence and cell type [44]. Therefore, initially designing a longer poly(A) tail may help maintain protein expression for a longer duration.

Chemical modification of the poly(A) tail, such as phosphorylation, can improve mRNA stability and prevent excessive degradation. For example, Strzelecka D et al. used gel electrophoresis to analyze the effect of phosphorylated poly(A) tails on the deadenylation of mRNA by recombinant human CNOT7. They found that, in the presence of ATP, mRNA polyadenylated by PAP is significantly less sensitive to deadenylation, with the degradation rate correlating with the modification frequency (the higher the thio-phosphate content, the more stable the poly(A) tail). Furthermore, in HeLa and JAWS II cell lines, mRNAs with various modified poly(A) tails showed that thio-phosphate modification did not affect protein expression, regardless of content [45].

Compared with continuous poly(A) sequences, segmented poly(A) tails can greatly reduce plasmid recombination rates and significantly boost both protein translation levels and mRNA yield. Trepotec Z et al. tested three types of plasmids for recombination in E. coli and found that the recombination rate for the poly(A)120 plasmid reached 50%, whereas rates for poly(A)2 × 60_6 and poly(A)3 × 40_6 plasmids significantly dropped, with poly(A)2 × 60_6 falling below 20%. Moreover, when transfecting A549 cells with mRNAs featuring different segmented poly(A) tails, protein expression and mRNA yield at 4- and 24-hours post-transfection were significantly higher than for the poly(A)120 control [46]. Recent research has shown that using multi-tailed mRNA can markedly increase translation efficiency. Chen Hongyu et al. enzymatically synthesized mRNAs with multiple poly(A) tails using a 30 nt poly(A) RNA chain as a branch, connected the product to a firefly luciferase (FLuc) reporter gene, and found that the optimized multi-tailed mRNA produced intracellular luminescence signals 4.7–19.5 times higher than the control mRNA 24–72 h after transfection [47].

#### Modified nucleotides

**Fig. 5:**
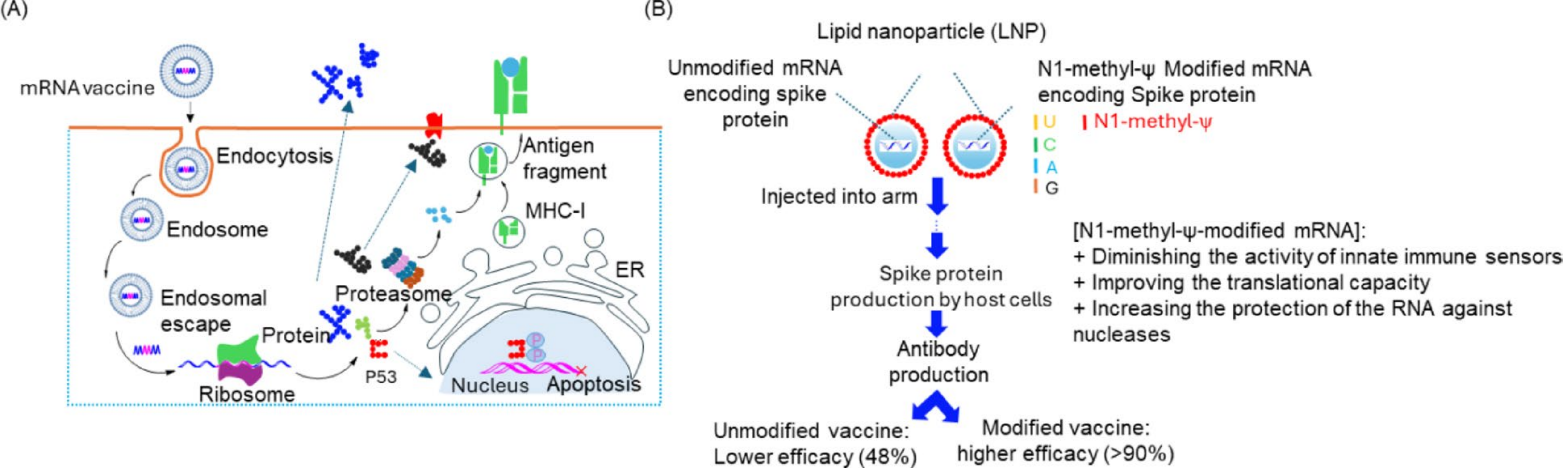
(**A**) scheme showing the intracellular delivery and translation process of mRNA encapsulated in the LNP. (**B**) Different mRNA vaccine efficacy using pseudouridine (ψ) or N1-methylpseudouridine (m1ψ)

As the essential template for target protein synthesis, the coding sequence (CDS) benefits greatly from nucleotide modifications, which can effectively prevent recognition of mRNA by pattern recognition receptors (PRRs) and significantly reduce the negative regulation of adaptive immunity by type I interferon (IFN-I) responses [48] (Fig. 5)**.** In 2005, groundbreaking research by Katalin Karikó and Buckstein M demonstrated that introducing pseudouridine into the RNA sequence could reduce its immunogenicity, with immunogenicity decreasing proportionally as the pseudouridine content increased [49]. Furthermore, mRNA in which uridine is entirely replaced by pseudouridine not only dramatically reduces immunogenicity but also increases stability and enhances translational capacity [50]. Subsequent studies have shown that replacing uridine with pseudouridine (ψ) or N1-methylpseudouridine (m1ψ) can significantly reduce innate immune stimulation while simultaneously enhancing mRNA translation efficiency (Fig. 5B). This breakthrough not only solved the core challenge of mRNA immunogenicity but also played a pivotal role in therapeutic mRNA and vaccine development, ultimately earning Katalin Karikó and Drew Weissman the 2023 Nobel Prize in Physiology or Medicine [51].

**Fig. 6:**
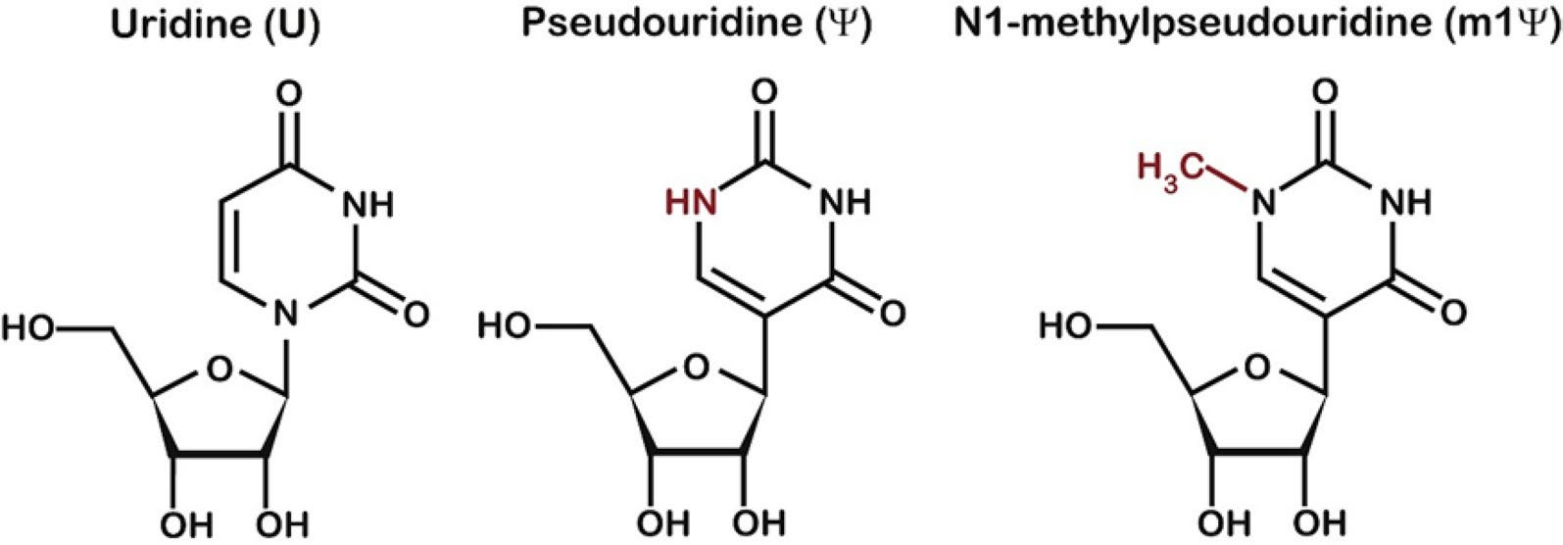
Chemical structures of uridine and its modifications

Currently, modifications based on pseudouridine (Ψ) and N1-methylpseudouridine (m1Ψ) are predominantly used in mRNA vaccines and therapeutics. Oliwia Andries et al. conducted comprehensive in vitro transfection and in vivo experiments using mRNA molecules containing different modifications—N1-methylpseudouridine (m1Ψ) alone, pseudouridine (Ψ) alone, or a combination of both. Their results demonstrated that m1Ψ-modified mRNA offered higher protein expression levels and lower cytotoxicity and innate immune stimulation in mammalian cell lines and mice compared with Ψ-modified mRNA [52]. Similarly, Kyusik Q. Kim et al. used a cell-free translation system, in vitro assays, and RNA duplex stability studies to investigate the effects of m1Ψ and its related modified nucleotide Ψ. Their findings showed that m1Ψ does not significantly affect translational fidelity [52, 53]. (Fig. 6) These studies collectively indicate that N1-methylpseudouridine (m1Ψ) demonstrates superior effects on mRNA modification compared to pseudouridine (Ψ). However, recent research has revealed potential concerns regarding continuous incorporation of N1-methylpseudouridine. Studies suggest that extensive m1Ψ modification may induce ribosomal + 1 frameshifting, potentially triggering T cell immune responses to frameshift-derived peptides [54]. his finding highlights the critical necessity of thoroughly evaluating the functional effects of new modified nucleotides from multiple perspectives during mRNA development to ensure both safety and efficacy.

### Purity and impurity control

**Fig. 7:**
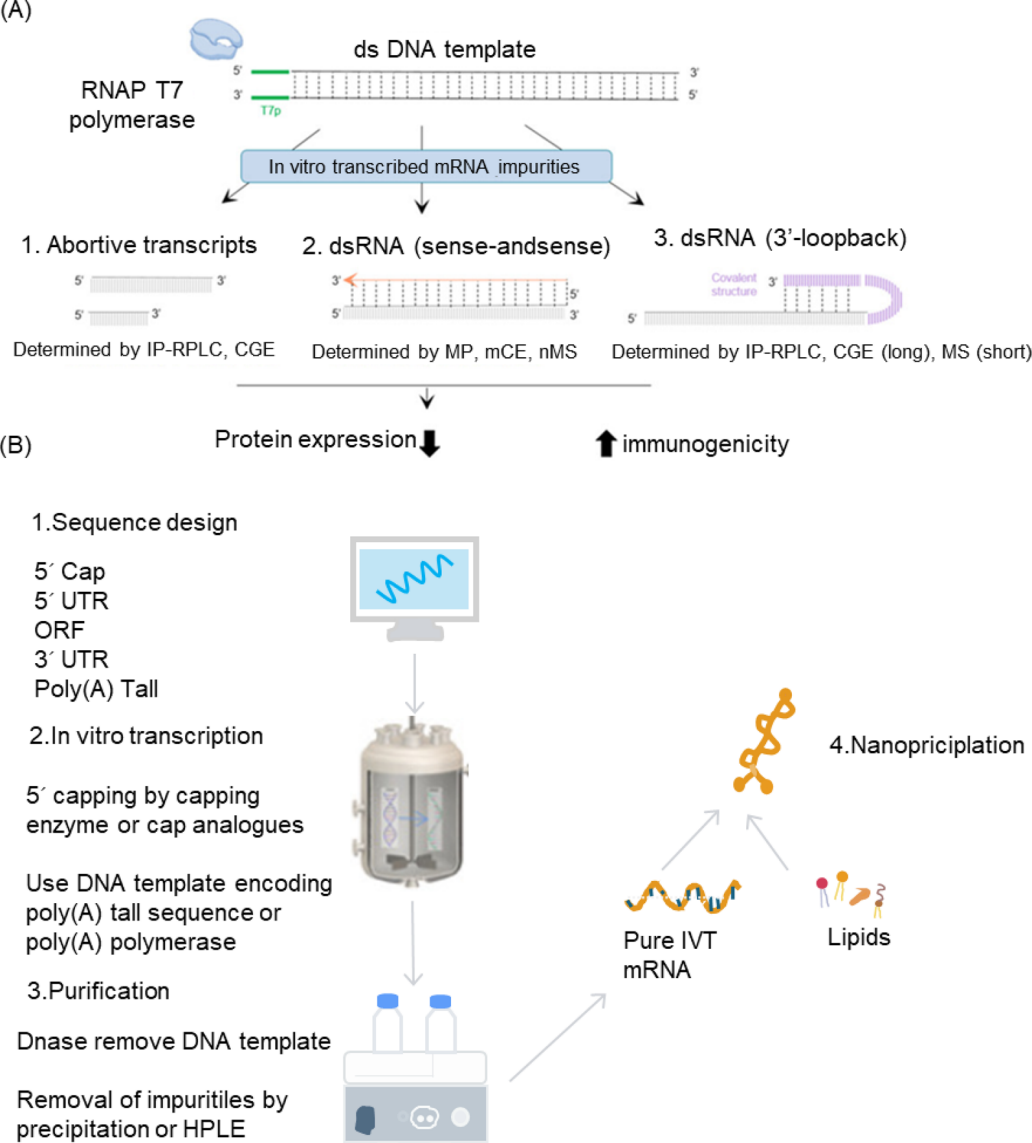
(**A**) The Impact of In Vitro-Transcribed mRNA Impurities on Cellular Responses (**B**) Schematic illustration on the key production process of the IVT mRNA and its lipid-based formulation

In vitro synthesis of mRNA uses plasmid DNA (pDNA) as a template, with pDNA containing an RNA polymerase promoter (such as T7) and the mRNA template sequence. Under the catalysis of RNA polymerase (e.g., T7 phage RNA polymerase, T7 RNAP), pDNA, together with nucleotide triphosphate substrates (NTPs), enables high-fidelity in vitro synthesis of mRNA. During or after the transcription process, 5’ capping and 3’ poly(A) tailing are also performed enzymatically [55, 56] (Fig. 7A).

In vitro mRNA synthesis introduces various materials that may potentially remain as residues in the final product, including plasmid DNA, RNA polymerase, metal ions, and nucleotides. Materials generated through microbial fermentation, such as plasmids and enzymes, may also contain process-related impurities, including host cell DNA, host RNA, host proteins, and endotoxins [56, 57] (Fig. 7B)**.** Additionally, due to the inherent complexity of in vitro transcription, mRNA-related impurities are inevitably produced. The most notable impurity is double-stranded RNA (dsRNA), along with fragmented RNAs (including uncapped RNAs, transcription termination fragments, and degraded RNAs), and RNA: DNA hybrids [58–63].

Generally, mRNA produced by in vitro transcription (IVT) may contain the following categories of impurities: Linear DNA templates (linearized plasmids or PCR products), nucleotide triphosphates (NTPs), cap analogs, and RNA polymerase (RNA Pol II); IVT byproducts and their derivatives; RNase, endotoxins, metal ions, and solvents introduced via raw materials and production processes [59].

#### Purity and fragmented RNA

During the IVT process, short mRNA fragments ranging from 2 to 13 nucleotides may be produced as byproducts [64–66]. These single-stranded RNA (ssRNA) fragments, along with other ssRNA species, can activate toll-like receptors (TLR) 7 and 8, thereby inducing the release of type I interferons. Similarly, the presence of double-stranded RNA (dsRNA) can activate inflammatory signaling pathways, including NF-κB, RIG-1, and MDA5 [58, 67]. mRNA degradation, particularly through hydrolysis, can generate additional fragmented RNA species and reduce overall product purity. This degradation process is significantly accelerated under alkaline conditions and in the presence of RNase and metal ions (e.g., Mg²⁺) [68–75].

Compared to their full-length counterparts, impure or fragmented mRNAs lack essential structural elements required to maintain mRNA stability and expression. These critical structures include the 5’ cap, full-length poly(A) tail, and untranslated regions (UTRs), the absence of which results in decreased stability and reduced translation levels. Furthermore, the presence of multimeric mRNA can also compromise translation efficiency and may induce additional adverse effects, potentially impacting the overall therapeutic performance of the mRNA product [55, 58, 75–80].

#### DsRNA

Double-stranded RNA (dsRNA) represents a heterogeneous population of sequences of varying lengths produced by the abnormal activity of T7 RNA polymerase. While fragmented RNA negatively affects mRNA translation efficiency, dsRNA byproducts have an even greater impact on immune response immunogenicity. Consequently, dsRNA is currently the most extensively studied IVT byproduct [55, 60, 63, 81].

dsRNA formation occurs through three primary mechanisms: (1) Longer dsRNA byproducts are mainly generated when 3’-extended runoff products anneal to complementary sequences on the runoff transcript, or when normal RNA re-binds to the enzyme after transcription and is used as a template for extension to form dsRNA [62]; (2) T7 RNA polymerase initiates promoter-independent transcription using the non-template strand to form antisense RNA molecules, which subsequently anneal with normal transcripts to form dsRNA; (3) Abortive transcripts undergo random pairing to generate dsRNA structures [60–63, 81, 82].

In the body, various pattern recognition receptors (PRRs) sense dsRNA and trigger innate immune responses upon recognition, forming a sophisticated multi-layered sensing network. Retinoic acid-inducible gene I (RIG-I) primarily recognizes dsRNA with 5′-triphosphate (5′-ppp) or 5′-diphosphate (5′-pp) modifications, with optimal signaling occurring at lengths of 20–150 bp. Melanoma differentiation-associated protein 5 (MDA5) preferentially recognizes long dsRNA sequences over 1000 bp, with recognition that is independent of 5’ end modifications. TLR3, a member of the toll-like receptor (TLR) family, recognizes dsRNA longer than 40 bp and activates downstream interferon (IFN) and nuclear factor κB (NF-κB) pathways to initiate immune responses. Other dsRNA-binding proteins are also involved in innate immune regulation: protein kinase R (PKR), upon binding dsRNA of at least 33 bp, inhibits protein synthesis by phosphorylating eukaryotic initiation factor eIF2α and induces NF-κB–mediated apoptosis; 2′,5′-oligoadenylate synthase (OAS) activates RNase L after binding dsRNA, causing extensive degradation of cellular RNA [83]. Collectively, these diverse recognition mechanisms constitute a comprehensive, multi-layered dsRNA sensing and immune response network that enables rapid detection and response to foreign RNA species.

Innate immune responses and translational inhibition can seriously impair the safety and efficacy of mRNA vaccines. Techniques such as using engineered T7 RNA polymerase, optimizing transcription systems, adding urea during transcription, 5’ dephosphorylation of uncapped transcripts, RNase III digestion, cellulose removal of dsRNA, and chromatographic purification can effectively reduce dsRNA, prevent innate immune activation, and increase protein expression [55, 61, 64, 77, 79, 80, 84–88].

#### RNA: DNA hybrids

During the IVT process, newly synthesized RNA strands can displace the non-template strand from the DNA duplex and anneal to the template strand of the DNA, thereby forming stable RNA: DNA hybrids. Oligonucleotides generated by enzymatic digestion of plasmid DNA (pDNA) can also base-pair with portions of the transcript to form DNA–RNA hybrid fragments. Using T7 RNA polymerase for IVT on templates rich in purine sequences or containing multiple GAA repeats will generate large amounts of RNA: DNA hybrids [58, 89, 90]. RNA: DNA hybrids can activate innate immune signaling pathways and induce the expression of cytokines, chemokines, and type I interferons, thereby triggering unnecessary innate immune responses, and pose a risk of genomic integration [58, 59, 91–93].

#### Other residual impurities

In addition to IVT byproducts, various exogenous contaminants or impurities may be present, arising from raw materials or manufacturing processes. These include residual DNA, enzymes, nucleotides, metal ions, and endotoxins, each presenting distinct safety and efficacy concerns.

If residual DNA remains intact after administration and penetrates the cytoplasm, there may be a risk of genomic integration [57, 94]. Polymerases may be recognized as foreign antigens, inducing the release of pro-inflammatory cytokines as part of the adaptive immune response, and leading to inflammation [58, 95, 96]. Residual nucleotide triphosphates may bind to purinergic receptors (P2), activating neuroinflammatory pathways in the central nervous system [97]. RNase residues can cause the degradation of IVT mRNA, affecting its integrity and compromising its activity [70–72]. Residual divalent metal ions, such as Mg^2+^, may promote non-enzymatic degradation of mRNA and RNase-mediated degradation, thereby impacting integrity, stability, and activity [69–75]. Even endotoxin levels as low as 0.1–0.5 ng/kg can induce cytokine release and may result in dose-dependent adverse effects such as fever, chills, nausea, hypotension, tissue damage, sepsis, and death [59, 98, 99].

### Delivery system characteristics

**Fig. 8:**
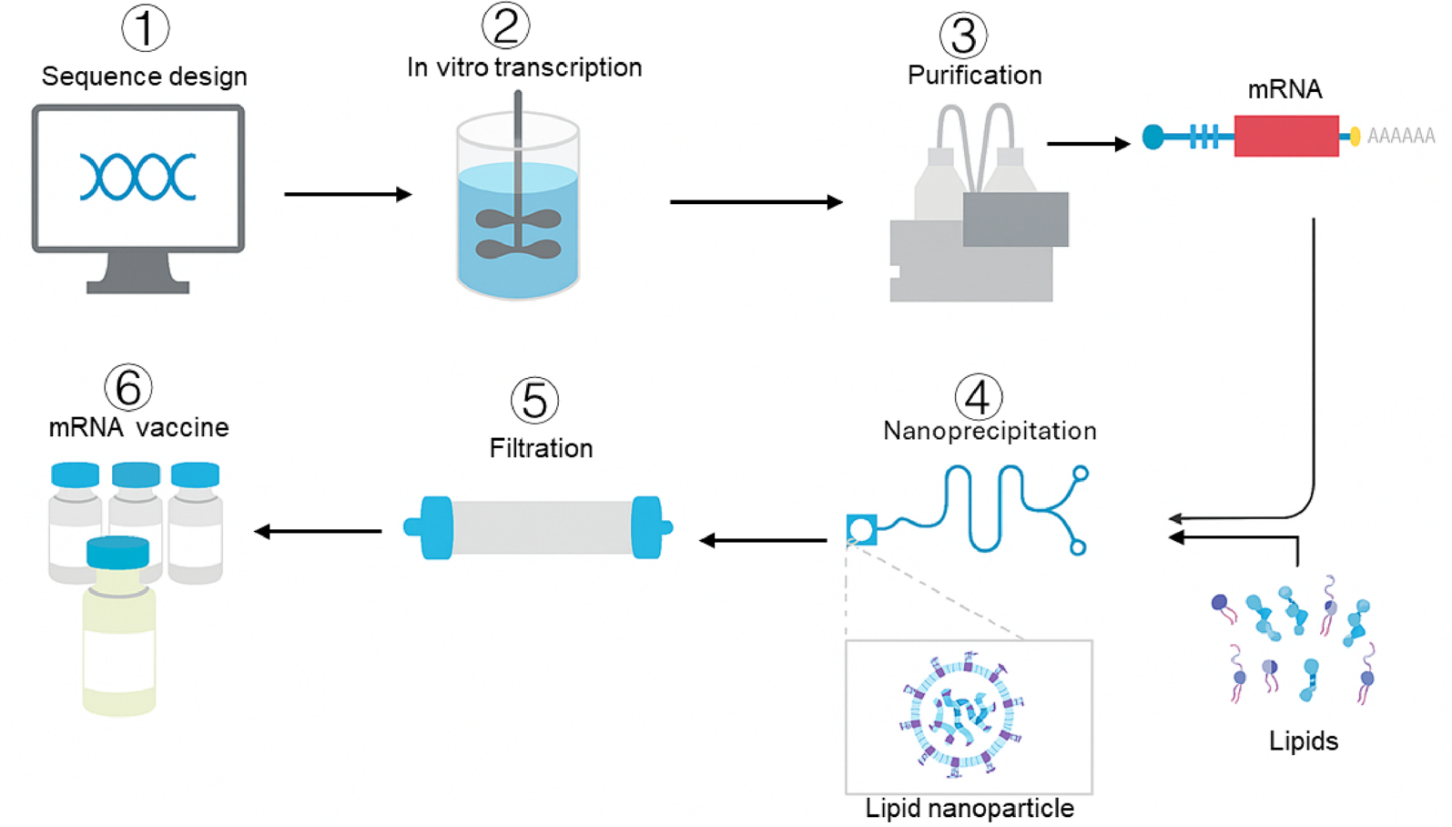
The mRNA is synthetically produced and formulated into vaccines

In recent years, various delivery systems have been developed and used for mRNA delivery, such as lipid nanoparticles (LNPs), polymeric nanoparticles (PNPs), and lipopolyplexes (LPPs) [100]. (Fig. 8)**.** Among these, LNPs are currently the mostly used delivery system for mRNA vaccines. LNP was initially developed for siRNA delivery. The first LNP-based siRNA drug, Onpattro, received FDA approval in 2018. During the COVID-19 pandemic, mRNA vaccines emerged as a breakthrough solution. Both COVID-19 mRNA vaccines selected LNP as their delivery platform, demonstrating the efficacy of the mRNA-LNP delivery system. In addition to COVID-19 vaccines, several other vaccines developed using the mRNA technology platform is currently in development, with most employing LNP as their delivery system [101]. The composition of LNPs is not fixed. Numerous lipid components have been developed to enhance LNP safety, targeting, delivery efficiency, and endosomal escape efficiency. Permanent cationic lipids like DOTMA were first developed for DNA delivery, but they may cause harmful cellular side effects. To address their cytotoxicity, ionizable lipids like DLinDMA and DLin-MC3-DMA were developed. However, their delivery efficiency remains limited—for instance, the FDA-approved DLin-MC3-DMA-based LNP mediates only 1–4% RNA release into the cytoplasm. In contrast, the ionizable lipids used in COVID-19 mRNA vaccines, ALC-0315 and SM102, demonstrate superior safety, stability, and endosomal escape efficiency compared to DLin-MC3-DMA. Development of LNP components remains an ongoing endeavor [102, 103]. A typical LNP consists of four types of lipids: ionizable lipids (e.g., ALC-0315, SM102), PEG-lipids (e.g., ALC-0159, PEG2000-DMG), helper lipids (e.g., DSPC), and cholesterol. Ionizable lipids, PEG-lipids, and helper lipids all contain hydrophobic tails, which aggregate via hydrophobic interactions to form the LNP core. In an acidic environment, the head groups of ionizable lipids become protonated and positively charged, enabling electrostatic binding with negatively charged mRNA molecules to ensure effective encapsulation of nucleic acids within the particles. PEG-lipids are distributed on the LNP surface; their hydrophilic head groups reduce particle aggregation and stabilize dispersion through steric hindrance. Helper lipids influence the structural stability of LNPs. Cholesterol fills gaps between lipid molecules, affecting lipid fluidity. By mixing mRNA and the four lipids under acidic conditions, all components, driven by hydrophobic interaction, self-assemble into LNPs that encapsulate mRNA internally. The background of the publicly disclosed patent US8058069B2 by PROTIVA BIOTHERAPEUTICS INC indicates that researchers synthesized a delivery system for nucleic acid molecules such as siRNA and miRNA using ionizable lipids, PEG lipids, helper lipids, and cholesterol in varying composition ratios. The lipid composition ratios differed significantly across different implementation schemes, with the molar proportion of ionizable lipids ranging from 25% to 70% and PEG lipid molar proportions ranging from 1% to 4%. By delivering siRNA molecules to mice using this system and observing corresponding gene silencing activity to evaluate nucleic acid delivery efficiency, it was found that the implementation with 57.1% ionizable lipids exhibited optimal nucleic acid delivery efficiency. Additionally, multiple implementations with ionizable lipid proportions exceeding 50% also demonstrated favorable nucleic acid delivery efficiency. Based on this finding, the patent claims a nucleic acid delivery system composed of approximately 50% to 85% ionizable lipids, approximately 13% to 49.5% non-ionizable lipids, and approximately 0.5% to 2% PEG lipids. This patent represents a foundational patent in the LNP field. The LNPs used in the marketed products SPIKEVAX^®^ and COMIRNATY^®^ fall within the scope of protection of this patent [104]. As shown in Table 1, the commercial mRNA vaccine formulations approved by the FDA (including SPIKEVAX^®^ and COMIRNATY^®^) have similar LNP compositions and formulations [105–107].

Studies have shown that within an ionizable lipid proportion ≤ 50 mol%, the intensity of protein expression from mRNA in mice increases with the proportion of ionizable lipids, but exceeding this threshold instead leads to reduced protein expression, possibly due to increased clearance rates [108]. Among helper lipids, cholesterol (with a representative proportion of 38.5 mol%) stabilizes the lipid bilayer structure, thereby enhancing LNP integrity and membrane fusion capability. Phospholipid DSPC (accounting for 10 mol%) simulates natural membrane components and effectively promotes cellular uptake of LNPs. Although PEGylated lipids are present in the lowest proportion (< 5 mol%) in the LNP system, their content, hydrophobic chain length, and end group structure markedly affect the stability and biological activity of LNPs: high PEG content may reduce delivery efficiency due to steric hindrance affecting RNA release, while short-chain PEGs (e.g., C14) can improve transfection efficiency but may reduce serum stability [108–110]. Research has shown that different types of helper lipids and PEG lipid ratios can influence long-lasting immune responses. LNPs formulated with DSPC phospholipids as the helper lipid and 0.5% PEG lipid demonstrated stronger long-term immune induction efficacy compared to LNPs using DOPE as the helper lipid or those with a 1.5% PEG lipid content [111]. This suggests that helper lipids and PEG lipids not only regulate the physicochemical properties of LNPs but also influence their adjuvant effects, thereby modulating the immunogenicity and safety of mRNA vaccines. In summary, precise control over the formulation ratios of each component is of great significance to the rational design and product development of mRNA vaccines.

**Table 1:** LNP formulations of *SPIKEVAX*^®^ and *COMIRNATY*^®^

Formulation		Trade name	
		SPIKEVAX^®^	COMIRNATY^®^
Lipid Components	Ionizable Lipid	SM-102	ALC-0315
	PEG Lipid	PEG(2000)-DMG	ALC-0159
	Helper Lipid	DSPC	DSPC
	Cholesterol	Cholesterol	Cholesterol
Lipid Molar Ratio		50.0 : 1.5 : 10.0 : 38.5	47.3 : 1.7 : 9.6 : 41.4
N/P Ratio		5.4	6.3

The composition and formulation of LNPs affect their physicochemical properties such as particle size and surface charge, which in turn influence the targeting, safety, and expression efficiency of LNPs.

#### Lipids

Among the four classic basic components that constitute the LNP carrier, cholesterol and DSPC are conventional ingredients that have been widely used in liposomal drugs and are registered in the FDA Inactive Ingredient Database (IID); ionizable lipids and PEG-lipids, as innovative components, often vary in mRNA vaccine products depending on product type and/or manufacturer and are usually novel molecular entities. Both are core components of the mRNA vaccine delivery system, playing a dual role in maintaining structural stability and regulating functional properties. Ionizable lipids and PEGylated lipids affect critical properties such as mRNA encapsulation efficiency, immunogenicity, and formulation stability through different mechanisms.

Ionizable lipids can be divided into three regions: head group, linker, and hydrophobic tail. The head group is a pH-dependent ionizable group that usually influences the surface charge of the LNP, thereby affecting its immunogenicity, tissue distribution, etc. The linker can be categorized into two major types based on metabolic stability: non-biodegradable (e.g., ether, carbamate) and biodegradable (e.g., ester, amide). Non-biodegradable types can enhance transfection efficiency but may increase cytotoxicity due to their stability and poor clearance; biodegradable types are usually cleared quickly in vivo and thus present fewer adverse effects. The hydrophobic tail is mainly composed of alkyl chains, and its length and degree of unsaturation influence hydrophobic interactions, affecting the fluidity and rigidity of LNP structure, which in turn impacts LNP encapsulation efficiency and transfection efficiency [112, 113]. In addition, since the tails of PEG-lipids and helper lipids are also alkyl chains, structural adjustments of these tails can similarly affect LNP immunogenicity. Studies have shown: (1) Specific cationic lipids (such as DOTAP) can activate Toll-like receptors and induce inflammatory responses, while proprietary ionizable lipids can act as adjuvants via IL-6 but do not rely on Toll-like receptors [114]; (2) Administration via intramuscular injection in mice revealed that immunogenicity is highly dependent on lipid structure, with the optimal pKa for immunogenicity being 6.6–6.9 (higher than the optimal pKa for transfection, 6.2–6.5), and animal studies show that protein expression levels are not necessarily correlated with immunogenicity. However, it should be noted that different species may exhibit significant variations in their response to LNPs: cellular heterogeneity influences the in vivo behavior of mRNA-LNPs with different components; cellular metabolic states affect LNP delivery efficiency; and the activation and deactivation of inflammatory signaling pathways impact mRNA translation processes [115]. Thus, when studying LNP delivery systems, attention should be paid to species and cell type differences [116]. Therefore, in mRNA vaccine development, in addition to meeting conventional excipient requirements such as structure and purity, it is also necessary to comprehensively evaluate the safety (e.g., degradability, metabolic clearance pathway, compound interactions) and efficacy (e.g., in vitro antigen expression, in vivo delivery efficiency, immune response intensity) parameters of ionizable lipids, and to optimize key parameters such as hydrophobic branch chain, charge distribution, and particle size based on structure–activity relationships to balance delivery efficiency and safety.

**Fig. 9:**
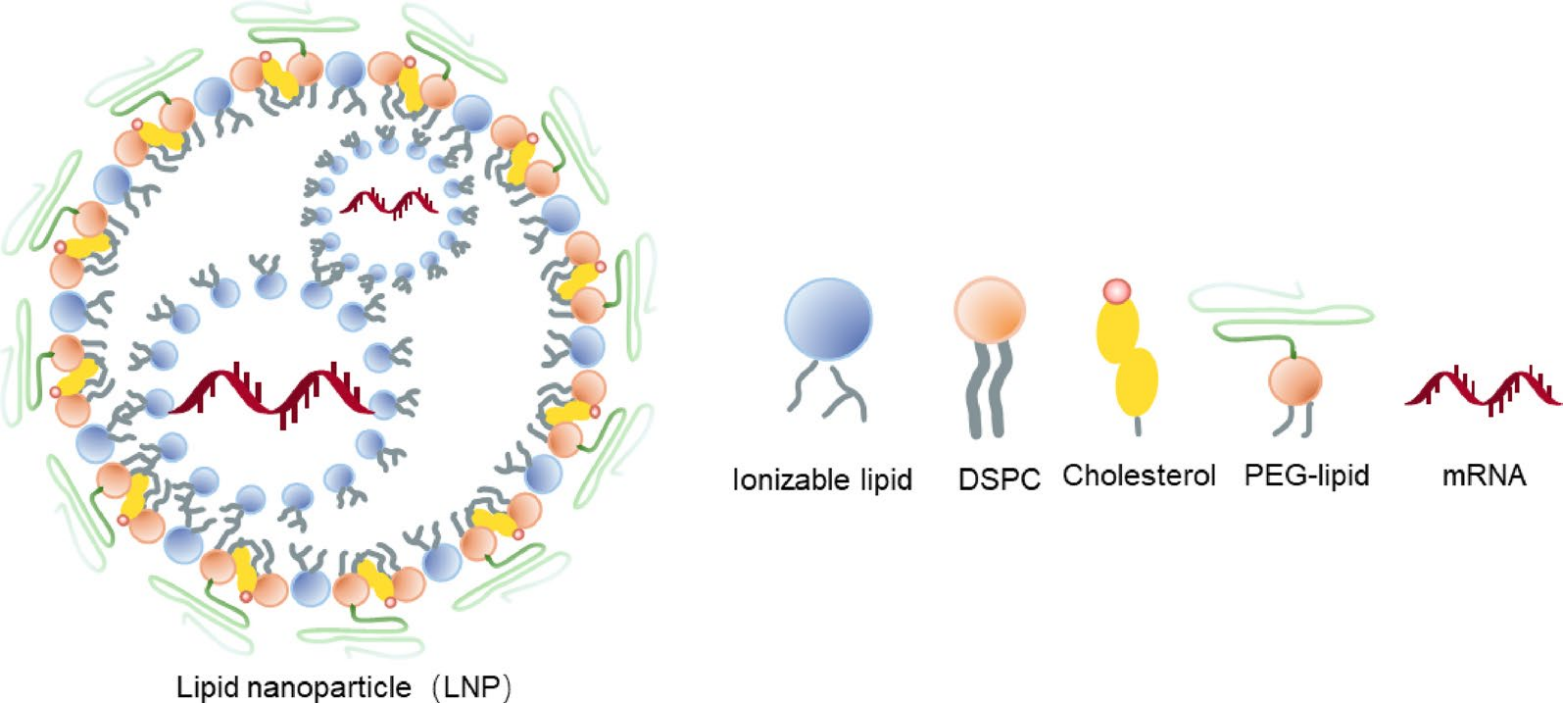
Simplistic illustration of LNP and its individual components

PEG-lipids are composed of hydrophobic lipid tails covalently linked to hydrophilic PEG chains. They enhance nanoparticle stability and prolong circulation time through steric hindrance but may also affect cellular uptake. (Fig. 9) For example, in the LNP delivery system of Pfizer’s COVID-19 vaccine Comirnaty, studies in Wistar rat models have shown that repeated injections induce a dose-dependent production of anti-PEG IgM/IgG antibodies and accelerate clearance of LNPs from the blood [117]. PEG-lipids with different terminal groups show varied immune responses: those with hydroxy end-groups induce lower anti-PEG antibodies but stronger complement activation, whereas mainstream formulations (such as mRNA-1273, BNT162b2) use methoxy end-groups to balance safety and efficacy [118]. In addition, parameters such as the hydrocarbon chain length, branched structure, and surface density of PEG-lipids influence complement activation and the ABC (accelerated blood clearance) effect, thereby determining the product’s safety profile [110].

Thus, in mRNA vaccine development, attention should be paid to PEG chain length (e.g., 2 kDa ~ 5 kDa), linker type (ester/amide bond), degree of polymerization distribution, and terminal group structure. These factors can significantly affect the formulation’s immunogenicity (including anti-PEG antibody risk) and in vivo behavior (such as LNP stability and circulation time). Comprehensive evaluation of PEG-lipid structural characteristics is needed to optimize formulation performance and reduce potential risks.

Currently, researchers are also exploring additional phospholipid materials beyond DSPC and DOPE. For instance, novel helper lipids designed by Gomi et al. enable targeted delivery of LNPs to the spleen and secondary lymphoid tissues, while Liu et al. enhanced mRNA delivery efficiency by screening ionizable phospholipids [102, 119, 120].

By modifying existing lipids or developing novel lipid structures, LNP performance can be further improved. For instance, integrating ionizable amine groups and polyalkyl chains into phospholipid structures has been shown to enhance mRNA endosomal escape efficiency [120]. Transforming low-cost commercial cationic polymers into phospholipidated and alkylated polymers (PAPs), enables efficient and organ-selective mRNA delivery in vivo [121]. Incorporating thiolated DSPE into LNP formulations enables binding to cystine domains in the bladder, facilitating bladder-targeted therapies. Development of novel lipid components and LNP optimizations remain ongoing. Predicting lipid structures, process parameters, and lipid composition ratios using machine learning tools represents another major research direction. For instance, Ravi et al. employed the SVEM model to predict LNP characteristics, focusing on evaluating particle size, PDI, zeta potential, heat trend cycle, encapsulation efficiency (EE), recovery ratio, and encapsulated mRNA content. The reliability of their SVEM model predictions was validated through experimental results [122].

#### Physicochemical properties of LNP

By regulating physicochemical properties such as particle size and PDI, surface charge, and morphology, the targeting, safety, and expression efficiency of mRNA vaccines can be influenced.

##### Particle size and PDI

Particle size refers to the average diameter of LNP particles and is commonly characterized by the hydrated hydrodynamic radius measured through dynamic light scattering (DLS). PDI (polydispersity index) is an indicator of the uniformity of LNPs and ranges from 0 to 1; the smaller the value, the more concentrated the particle size distribution and the higher the uniformity; the larger the value, the broader the particle size distribution and the less uniform the particles.

**Fig. 10:**
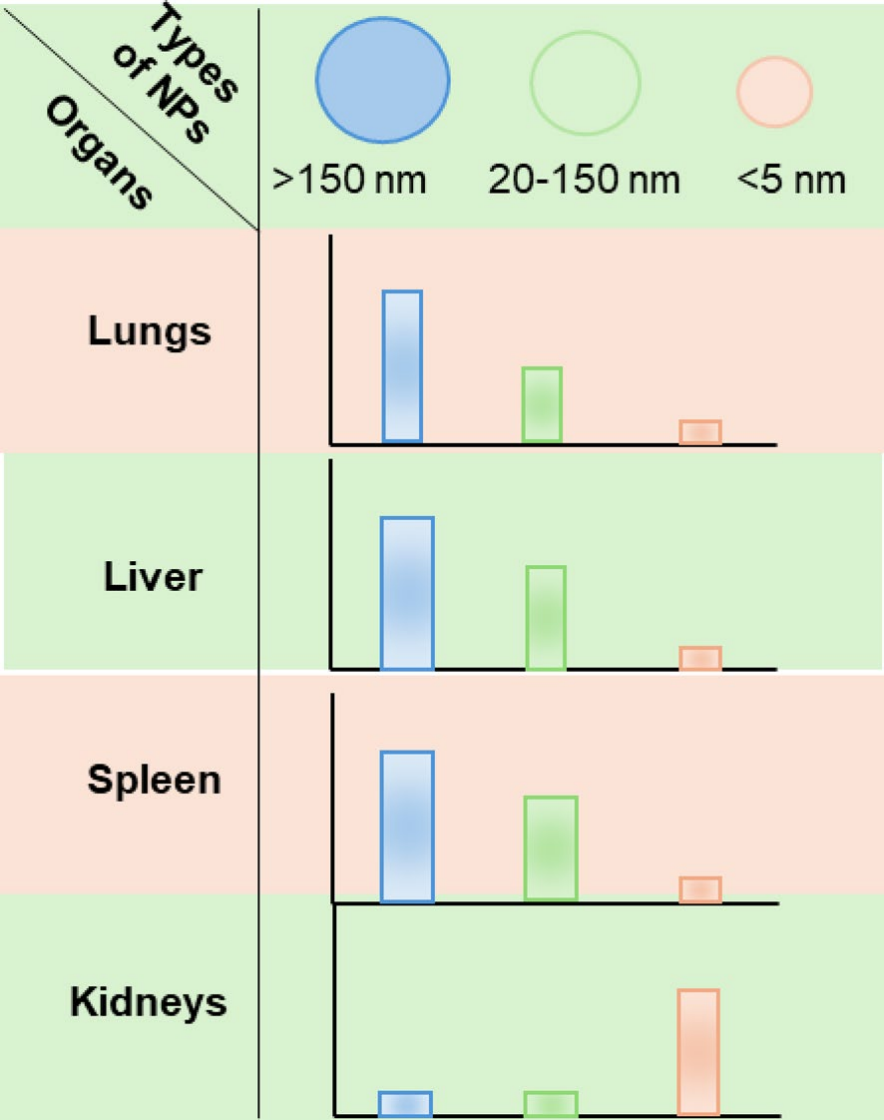
The size, shape and surface charge of NPs

Particle size affects the lymphatic system targeting, cellular uptake efficiency, tissue distribution, and expression efficiency of LNPs. Studies have shown that nanoparticles sized 20–200 nm can freely diffuse into lymph nodes and have good lymphatic targeting. In subcutaneous injection experiments in mice, nanoparticles of this size can more efficiently drain and penetrate lymph nodes; nanoparticles larger than 200 nm depend on active transport by dendritic cells (DCs) and mostly remain at the injection site due to their size limitations during subcutaneous injection [123, 124]. In in vitro experiments on DC uptake of nanoparticles, larger particles have shown an advantage in cellular uptake efficiency, especially among antigen-presenting cells (APCs) [124]. Some research teams have injected LNPs of different sizes into mouse muscle and found that particle size affects tissue distribution of LNPs: in addition to local retention, LNPs of about 100 nm are mainly distributed in the liver with a small fraction in the spleen, 200 nm LNPs are also largely found in the liver, and those around 330 nm largely remain at the injection site. The same study found that 200 nm LNPs have an expression efficiency advantage over 100 nm LNPs in the liver [125]. Notably, different species show significant differences in sensitivity to LNP particle size. For instance, cynomolgus monkeys respond well to LNPs sized 60–150 nm, while mice respond best to LNPs sized 80–100 nm. These differences may be related to a more developed lymphatic system in primates [116, 126]. (Fig. 10)

##### Surface charge

**Fig. 11:**
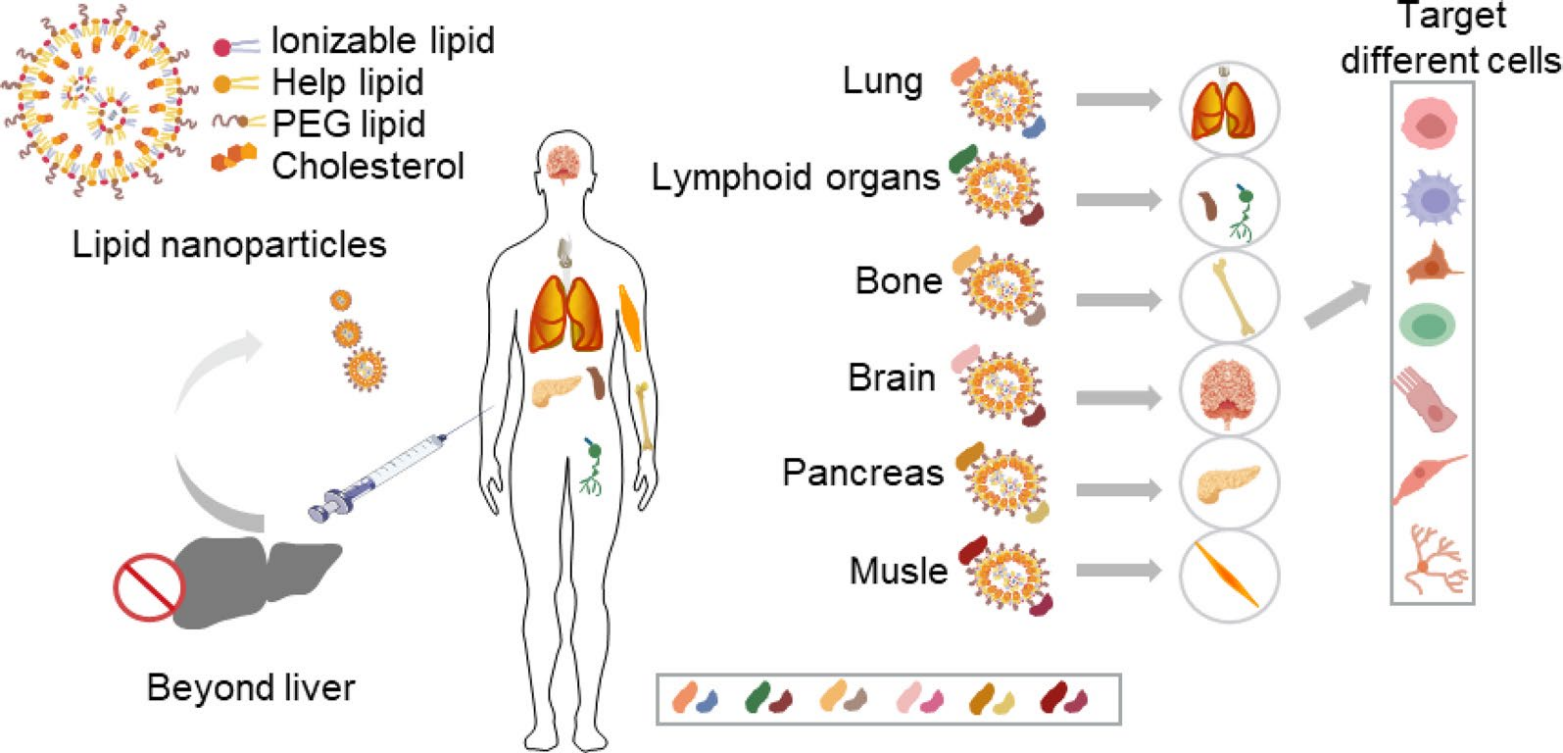
Schematic diagram of RNA-LNPs targeting extrahepatic organs

Surface charge affects the immunogenicity, tissue distribution, and payload release efficiency of LNPs. Charge characteristics can be systematically characterized by monitoring zeta potential, pKa, and isoelectric point (pI): zeta potential reflects the effective charge at the particle-medium interface, and systems are generally considered stable when the absolute value is >30 mV, although zeta potential is regulated by factors such as medium pH, ionic strength, and surfactants [127]. The effects of charge are mainly manifested in the following aspects: (1) Safety: Neutral systems usually exhibit optimal safety; LNPs with positive or negative surface charges tend to bind non-specifically with proteins and cells in circulation, promoting active uptake by macrophages and scavenger endothelial cell receptors. Positively charged LNPs can interact with the negatively charged surfaces of the glomerular basement membrane and podocytes, thus accelerating renal clearance [128, 129]; (2) Stability: High zeta potential inhibits particle aggregation via electrostatic repulsion [130]; (3) In vivo distribution: Neutral LNPs are targeted to the liver through APOE3 mediation, positively charged particles tend to accumulate in the lungs, and negatively charged particles preferentially enrich in the spleen [131] (Fig. 11); (4) Delivery efficiency: In acidic environments (pH ≈ 4), protonation of ionizable lipids promotes mRNA encapsulation; at physiological pH (7.4), weakly negative charge prolongs circulation time; and endosomal acidification (pH ≈ 6) triggers reprotonation, membrane fusion, and cytoplasmic release. This pH-responsive charge-switching mechanism is one of the core principles enabling efficient LNP delivery [132]. Most LNPs achieve liver targeting and enrichment, while extrahepatic targeting LNP technologies remain urgently needed. Regulating LNP surface charge to enable extrahepatic delivery is a major current research direction. Studies indicate that: Modulating the surface charge of LNPs by adjusting the lipid-to-mRNA mass ratio or incorporating additional lipid components can regulate delivery targeting. This enables targeted delivery to extrahepatic organs such as the lungs and spleen, as well as to immune cells, thereby expanding the application scope of LNP technology [115].

##### Morphology

Although mRNA-LNP COVID-19 vaccines have achieved global success, their nanoscale structures are still not fully understood, and their different morphologies affect LNP delivery efficiency and cytotoxicity. Transmission electron microscopy (TEM) offers unique advantages for the observation of mRNA-LNPs, allowing high-resolution direct visualization of structural information to intuitively obtain and quantify morphological features, size, and particle distribution of samples [133]. Studies based on TEM and other methods have shown that ultrastructural characteristics of mRNA-LNPs—such as core-shell distribution, surface topography, and asymmetric lipid arrangement—are key breakthroughs for understanding their delivery efficiency [134–136]. These nanoscale structural differences significantly influence the biological behavior of LNPs. For example, structural variations induced by manufacturing processes can cause LNPs with the same composition to present different structural characteristics and transfection efficacy [136]. Although cryo-EM yields intuitive results for mRNA-LNP structure determination, the equipment is not readily available, the tests are costly, and the detection process may disturb the sample structure, leading to limited result stability for LNP samples.

##### Encapsulation efficiency

Unprotected mRNA is easily degraded by nucleases and may also trigger excessive immune responses in vivo. Encapsulation of mRNA by LNPs enhances the stability and safety of the vaccine and is fundamental to the efficacy of mRNA vaccines. However, higher encapsulation efficiency may also indicate a more compact LNP structure, which could adversely affect the release of the mRNA payload. Under the premise of ensuring expression efficiency, high encapsulation efficiency can effectively protect mRNA from nuclease degradation and enable precise control of the dosing to improve antigen expression efficiency. Encapsulation efficiency of mRNA vaccines can be monitored using methods such as the Ribogreen fluorescent dye assay and ion-exchange chromatography. The Ribogreen fluorescent dye method is highly sensitive and easy to operate and is the current standard method for measuring mRNA-LNP encapsulation efficiency; it is also the USP-recommended assay for encapsulation rate monitoring of mRNA drugs. However, this method may be influenced by RNA fragment size, may have limited sensitivity when detecting complex samples, and cannot distinguish between completely encapsulated, surface-bound, and loosely encapsulated states of mRNA.

## Evaluation indicators of vaccine immunogenicity

Vaccine quality is a critical factor determining its protective efficacy and safety, affecting antigen presentation efficiency, strength of innate immune activation, as well as the polarization and activation level of adaptive immunity. In addition, differences in vaccine formulation composition and quality attributes may alter the targeting sites of vaccines, thus influencing mRNA release efficiency and cell-type specificity, ultimately resulting in differences in immune response activation in different tissues or organs.

### Innate immune responses activated by mRNA vaccines

mRNA vaccines activate the innate immune system through the LNP delivery carrier and mRNA molecules, with activation mechanisms primarily relying on pattern recognition receptors (PRRs) that recognize nucleic acid components [137, 138]. Specifically, ionizable lipids in LNPs (such as ALC-0315) can promote the uptake of mRNA by dendritic cells (DCs), thereby activating TLR4 or inflammasomes (such as NLRP3) and promoting the release of IL-1β and IL-6 [114, 139]. Simultaneously, unmodified single-stranded mRNA can be recognized by endosomal TLR7/8, triggering the secretion of type I interferons (IFN-α/β), which drives DC maturation and antigen presentation [5, 140]. However, nucleotide modifications (such as N1-methylpseudouridine) can reduce excessive inflammatory responses by lowering TLR affinity, while still maintaining translational efficiency [49, 50]. In addition, small amounts of double-stranded RNA (dsRNA) byproducts generated during mRNA translation can be recognized by cytosolic receptors RIG-I/MDA5, further amplifying IFN signaling [61]. This innate immune activation acts as a “double-edged sword”: moderate activation facilitates Th1 polarization and the formation of memory T and B cells, but excessive activation may lead to transient fever or local inflammation (such as the risk of myocarditis in adolescents). Fortunately, by optimizing LNP components (e.g., adjusting lipid ratios), the intensity of innate immune responses can be precisely regulated to balance vaccine efficacy and safety. In view of this, it is necessary to assess innate immune responses activated by the vaccine, including analysis of cytokines and chemokines (such as IL-6, IL-1β, IFN-α/β, CXCL8, etc.) as well as key innate immune cells (macrophages, NK cells, DCs, etc.).

### Adaptive immune responses activated by mRNA vaccines

#### Humoral immune response

mRNA vaccines activate humoral immune responses by encoding pathogen-specific antigens. The core mechanisms involve B cell activation, germinal center (GC) reactions, and the generation of high-affinity neutralizing antibodies. It has been demonstrated that high-affinity neutralizing antibodies are critical indicators of the ability of mRNA vaccines to induce robust humoral immunity post-immunization. mRNA vaccines encoding the full-length hemagglutinin (HA) of the influenza virus, encapsulated in lipid nanoparticles (LNPs), can circumvent the inhibitory effects of maternal antibodies, thereby elicit specific antibody responses and provide more durable and potent immune protection than conventional influenza vaccines. Feldman et al. reported Phase I clinical trial results for two non-replicating mRNA vaccines targeting the H10N8 and H7N9 influenza viruses, confirming their tolerability in healthy adults and their capacity to elicit strong humoral immune responses. This study also highlighted the potential of mRNA vaccines to address highly variable pathogens.

During the global COVID-19 outbreak caused by SARS-CoV-2 in 2020, Moderna was the first to announce its mRNA candidate vaccine, mRNA-1273, targeting SARS-CoV-2, and officially commenced Phase I clinical trials on March 16, 2020, to evaluate its safety and immunogenicity. This vaccine encodes the spike (S) protein of SARS-CoV-2. Interim data indicated that mRNA-1273 exhibited overall good safety and excellent tolerability. Two weeks after the second dose, even at doses as low as 25 µg, serum levels of binding and neutralizing antibodies were comparable to those found in convalescent COVID-19 patients. Simultaneously, Pfizer and BioNTech published ongoing Phase I/II clinical results for their vaccine candidate BNT162b1. This vaccine employs LNP-delivered modified mRNA technology. Following two doses of 10 µg and 30 µg, the mean titers of specific neutralizing antibodies were 1.8-fold and 2.8-fold higher than those in convalescent sera, respectively, underscoring the strong antibody response induced by mRNA vaccine immunization. Similarly, the Richner team developed a non-replicating mRNA vaccine encapsulated in LNPs encoding the human IgE signal sequence (IgEsig-prM-E) to prevent Zika virus (ZIKV) infection. After intramuscular injection of 2 µg followed by a booster immunization, extremely high titers of neutralizing antibodies (>1/100,000 EC_50_) were detected in mice [141].

Vaccine quality profoundly affects the immune outcome, determining the antibody titer, antibody affinity, neutralizing activity, and breadth, as well as temporal kinetics. This is particularly important when facing highly variable viruses such as influenza, HIV, or SARS-CoV-2. Studies on antibody breadth are crucial, as they comprehensively reflect the capability for neutralizing various pathogenic strains or subtypes. Currently, detection methods for neutralizing antibodies are mainly based on binding antibody detection and neutralizing antibody activity detection. For instance, in the analysis of the immunogenicity and efficacy of the self-amplifying mRNA ARCT-154 COVID-19 vaccine in phases 1, 2, 3a, and 3b, functional enzyme-linked immunosorbent assay and virus microneutralization assay were employed, respectively. These methods demonstrated acceptable safety and tolerability characteristics and showed immunogenicity when two doses of 5 µg or higher were administered 4 weeks apart [142]. In the interim analysis of SARS-CoV-2 variant mRNA vaccine boosters, Angela et al. used two pseudovirus neutralization methods, recombinant lentivirus PsVN assay and pseudovirus neutralization test, to measure the neutralizing antibody levels. They concluded that all boosters enhanced neutralizing antibody titers against key variants, with some variants showing higher titers, thereby demonstrating that the immunogenicity of the mRNA vaccine was significantly improved after booster vaccination [143].

For mRNA vaccines targeting respiratory syncytial virus prevention, immune responses were tested using competitive ELISA and neutralization assays. The neutralizing antibody titers in mice vaccinated with LC2DM-LNP were four times higher than those in mice vaccinated with mPre-F-LNPs, indicating that the former produced superior quality serum antibodies [144]. Additionally, in the development of adjuvanted mRNA vaccines, the introduction of complement protein C3 as a natural adjuvant increased anti-SARS-CoV-2 antibody titers by ten-fold compared to non-adjuvanted vaccines. Antibody titers were first detected through binding antibody titer assays, demonstrating that C3d-fused mRNA could induce high-level antibodies while reducing the dose to 0.1 µg. This was then verified by pseudovirus neutralization tests, showing that neutralizing antibody titers against SARS-CoV-2 Delta spike pseudotyped lentivirus could be significantly enhanced. Besides IgG, other antibody isotypes can also drive immune responses against SARS-CoV-2 in both mice and humans [145]. The development of high humoral immunity for mRNA vaccines extends beyond these examples. Other viruses, including respiratory viruses such as genital herpes virus, human cytomegalovirus, and rabies virus, all demonstrate significant binding antibody and neutralizing antibody responses [146]. In summary, this indicates that sera with typically high binding antibody titers simultaneously possess the ability to efficiently neutralize viruses in vitro.

Aside from antibody research, the activation of germinal centers (GCs) by vaccines must be emphasized. GCs are pivotal sites for B cell somatic hypermutation (SHM) and affinity selection [6, 147], and GC activation by vaccines is vital for the development of immune memory, improved antibody quality, and long-term vaccine protection. Stronger GC responses induced by vaccines yield higher-affinity neutralizing antibodies against pathogens (such as SARS-CoV-2) and increase the potential for cross-protection against variants [147]. Additionally, nucleotide modifications in mRNA vaccines lower TLR7/8-mediated innate immune activation, reducing inflammatory interference and optimizing GC response efficiency, thereby inducing broadly neutralizing antibodies and cross-protective memory B cells targeting conserved epitopes (such as those on Omicron variants) [50]. GC responses can be evaluated by flow cytometry phenotyping of B cells post-immunization, including GCBs, antigen-specific plasma cells, and memory B cells. Flow cytometric sorting can also obtain antigen-specific B cells, with single-cell PCR sequencing used to analyze SHM, clonal expansion, and diversity. Moreover, proliferation markers (such as Ki-67) and co-stimulatory molecules (CD40/CD86) are used to assess B cell activation status. The combination of these indicators is valuable for evaluating vaccine efficacy, for example, the persistence of memory B cells.

#### Cellular immune response

The cellular immune response activated by vaccines is primarily manifested as the activation, proliferation, and differentiation of antigen-specific T cells, especially CD4⁺ and CD8⁺ T cells. Studies have shown that the balance and specificity of CD4⁺ T cell responses are closely related to vaccine protective efficacy; they are essential, particularly for combating rapidly mutating pathogens, while imbalanced CD4⁺ T cell responses can even lead to vaccine-enhanced disease (VED) [148]. For example: local immune responses are triggered at the injection site through delivery into tissue-resident immune cells [149]. After booster immunization, broader and stronger inflammatory responses are observed, such as dramatic increases in inflammatory monocytes and IFN-γ [150].

Vaccine-activated CD4⁺ T cells coordinate adaptive immune responses through subtypes such as Th1, Th2, Th17, and follicular helper T cells (Tfh): Th1 cells secrete IFN-γ and IL-2 and are mainly responsible for antiviral and intracellular pathogen clearance; Th2 cells mediate antibody class switching via IL-4 and IL-5, potentially participating in allergic or anti-parasitic immunity; Tfh cells promote germinal center formation and high-affinity antibody production through IL-21 and CXCR5⁺PD-1⁺ markers, a key mechanism underlying the high protective efficacy of mRNA vaccines [7]. These responses not only enhance the synergy between humoral and cellular immunity, but also establish long-term immune memory. Therefore, in terms of cellular immune responses, mRNA vaccines currently employ flow cytometry to detect activation markers of CD4⁺ and CD8⁺ T cells (such as CD69 and CD107a), cytokine expression (such as IFN-γ, TNF-α, and IL-2), and cytotoxic molecules (such as granzyme B and perforin). This method evaluates T cell functional status and determines the Th cell subset preference of mRNA vaccines through cytokine expression levels. Enzyme-linked immunospot assay (ELISpot) is commonly used to detect the ability of individual cells to release cytokines (typically IFN-γ and IL-4) with high sensitivity, allowing for the evaluation of vaccine-induced T cell response frequency. MHC multimer staining utilizes MHC multimers to specifically bind to T cell receptors, directly detecting the frequency of antigen-specific T cells [151, 152].

In their study on optimizing LNP-mRNA vaccines, Li et al. detected a significant increase in IFN-γ secretion through ELISpot assay, indicating enhanced CD8⁺ T cell responses. They quantified dendritic cell activation and antigen presentation using flow cytometry, subsequently concluding a significant increase in CD4⁺ T cells. To evaluate the inflammatory response induced by the vaccine, they performed multiplex cytokine analysis on mouse serum and found that the optimized vaccine induced significantly lower systemic cytokine levels. This provides a comprehensive analysis and discussion of the cellular immune response elicited by the optimized vaccine [145].

Regarding the Th1 bias of T cell immune responses induced by mRNA vaccines, Goel et al. first demonstrated good cellular immune responses of the vaccine by measuring the substantial production of IFN-γ, IL-6, and IL-1β through RT-qPCR, and subsequently further confirmed the Th1 bias of mRNA vaccines by comprehensively measuring the quantity and types of cytokine-producing T cells through ELISA, intracellular cytokine staining (ICS), and ELISpot [144]. In contrast, vaccines with aluminum adjuvant can overactivate TLR2/4 and may induce IL-4/IL-5 production, thereby increasing the risk of allergies [153–155]. Therefore, detection of vaccine-activated memory T cells is crucial for evaluating long-term immune protection, with key indicators encompassing phenotypic markers, functional characteristics, and antigen specificity analysis. Infection challenge studies in animal models can determine whether vaccines can prevent or reduce disease symptoms or evaluate their protective efficacy in clinical trials by comparing infection rates, morbidity rates, and hospitalization rates between vaccinated and unvaccinated individuals [156, 157]. To verify the induction and maintenance of vaccine-specific immune responses, Goel et al. conducted clinical trials on 61 individuals who received either the Pfizer BNT162b2 or Moderna mRNA-1273 SARS-CoV-2 vaccines. They found that mRNA vaccination generated durable SARS-CoV-2-specific CD4⁺ T cell memory in individuals who had not been previously infected with SARS-CoV-2, but only transiently enhanced these responses in SARS-CoV-2 convalescents. In SARS-CoV-2-naive individuals, the magnitude and trajectory of all components of the immune response exhibited a consistent pattern shortly after the second dose. Finally, the assessment of the decay kinetics of SARS-CoV-2-specific recall responses revealed that mRNA vaccination could enhance infection-induced immune responses but did not significantly enhance existing memory B cell or memory T cell responses. Conversely, in the context of pre-existing immunity, the benefits of vaccination may be limited to significant but transient increases in antibody levels, with only a portion remaining after 6 months [158].

#### Mucosal immune responses activated by vaccines

Traditional injectable vaccines usually have limited ability to induce mucosal immunity, whereas vaccines administered via mucosal routes or designed with mucosal targeting can localize to mucosal tissues, driving the differentiation of antigen-specific B cells into IgA⁺ plasma cells to secrete sIgA, which neutralizes pathogens present on mucosal surfaces and offers rapid protection. Additionally, locally resident tissue-resident memory T cells (TRM) can provide rapid cytotoxic responses and cytokine-mediated (e.g., IFN-γ, IL-17) barrier defense [159, 160]. Studies have confirmed that intranasal COVID-19 vaccines can significantly enhance respiratory sIgA and TRM responses, providing cross-protection against Omicron variants [161]. Therefore, mucosal immune responses and their protective effects should be highlighted in vaccine immunoprotective studies.

### Modality comparison: mRNA vaccines vs. protein subunit vaccines

mRNA vaccines provide in situ antigen expression with native folding and post-translational modifications. This feature enables strong activation of T follicular helper (Tfh) cells and germinal center (GC) responses, leading to robust CD4⁺ Th1 and CD8⁺ T cell immunity. In addition, mRNA vaccines carry partly inherent adjuvanticity because RNA and lipid nanoparticle (LNP) components are recognized by pattern recognition receptors (PRRs). By contrast, protein subunit vaccines deliver exogenously produced antigens that generally require potent adjuvants to achieve comparable immunogenicity. While protein subunits can be conformationally optimized, they typically induce variable CD8⁺ responses and may lack intracellular processing signals.

The safety and reactogenicity profiles also differ between the two modalities. mRNA vaccines are associated with dose-dependent induction of innate cytokines, with rare myocarditis signals reported in specific demographics, and with risks of PEG-related hypersensitivity or accelerated clearance upon repeated dosing. These issues are closely tied to CQAs and formulation design. Protein subunit vaccines, on the other hand, often show adjuvant-driven reactogenicity, such as that arising from TLR agonists or alum, and hypersensitivity is usually linked to the adjuvant or excipient rather than the antigen itself.

Manufacturing considerations further highlight key distinctions. mRNA vaccines are template-agnostic, allowing rapid sequence replacement and leveraging highly automatable IVT and LNP processes. Although cold-chain requirements remain a challenge, advances such as lyophilized formulations are gradually reducing this burden. Protein subunit vaccines rely on cell-based expression, purification, and formulation systems, which are slower to redesign when new antigens are required, though they benefit from a more established cold-chain infrastructure.

From a standardization perspective, mRNA vaccines demand platform-specific CQAs, including dsRNA content, capping efficiency, and replicon integrity for self-amplifying constructs. In contrast, protein subunit vaccines emphasize CQAs such as protein identity, glycoform distribution, and aggregation control. Cross-modality, head-to-head studies using common immunological endpoints—including GC/Tfh readouts, T cell polyfunctionality, and neutralizing antibody breadth—are still limited. Such studies should be prioritized to clarify the unique advantages and disadvantages of each vaccine modality.

## Correlation analysis between CQAs and immunogenicity

### Gaps in current knowledge and standardization needs

Despite significant progress in the development of mRNA vaccines, important knowledge gaps remain, particularly regarding platform-specific quality attributes and assay harmonization. For conventional non-replicating mRNA, CQAs such as capping efficiency, RNA integrity, poly(A) length distribution, and dsRNA impurities are well recognized. However, standards for self-amplifying RNA (saRNA) remain unstandardized, despite its application in mRNA and peptide vaccine strategies against the COVID-19 causative agent, which incorporates multi-method B-cell epitope (BCE) prediction [162]. It includes replicon integrity, replication competence, and the presence and ratio of subgenomic RNA species. Furthermore, potency assessment for saRNA may require dual evaluation, capturing both early translation and later replication-driven antigen expression [163, 164]. Clear definitions and acceptance criteria for these attributes are lacking, which hinders comparability across studies and products.

Another critical gap lies in assay methods and reference standards. Although techniques such as RiboGreen encapsulation, dynamic light scattering (DLS), dsRNA ELISA or HPLC, and LC–MS for cap analysis are commonly employed, they are often used inconsistently across laboratories and manufacturers. At present, there are no universally accepted reference mRNA or LNP panels for method calibration. This lack of harmonization complicates cross-product benchmarking and makes it difficult to correlate specific CQAs with immunogenicity outcomes. Establishing shared assay standards and reference materials would represent a major step toward improving reproducibility and regulatory alignment.

Finally, gaps also exist in the translational immunology framework needed to link CQAs to immune responses. While several CQAs have been associated with innate immune activation, their downstream effects on antigen presentation, germinal center responses, and neutralizing antibody breadth remain incompletely defined. In particular, standardized panels to measure early innate cytokines, as well as consistent readouts for germinal center and Tfh cell activity, are not yet routinely applied. These tools will be essential for establishing causal links between product quality, immune response, and clinical efficacy across different mRNA platforms.

### Impact of mRNA quality on immunogenicity

Key quality parameters for RNA include integrity, purity, modified nucleotides, capping rate, and more. These critical quality parameters influence the antigen expression efficacy of the vaccine and the ensuing immune response in multiple ways. The inherent instability of mRNA means its integrity significantly affects vaccine efficacy. Pfizer’s research on the COVID-19 vaccine BNT162b2 showed that fragmented mRNA impurities lacking poly(A) cannot be translated into protein, demonstrating that mRNA integrity is essential for translational activity and vaccine efficacy [13]. Merck’s studies also confirmed a positive correlation between mRNA vaccine integrity and efficacy: increased temperature and prolonged time reduce integrity and vaccine efficacy, with efficacy approaching zero when integrity drops below 45% [165]. Clinical studies indicate a correlation between integrity thresholds and immune efficacy. Recent research has proposed that clinically effective mRNA vaccines should meet triple criteria: integrity ≥ 90%, supercoiled plasmid residue < 0.1%, and dsRNA residue < 0.01% [166]. Below this threshold, a 5% decrease in integrity corresponds to approximately a 50% reduction in neutralizing antibody titer [167]. Data from liver transplant recipients who received three doses of vaccine showed that seroconversion was 78% when vaccine integrity exceeded 95%, but only 42% when integrity was between 85% and 95% [167]. This suggests that immunocompromised populations are more sensitive to mRNA integrity, possibly due to lowered antigen presentation efficiency from impaired innate immunity. Furthermore, stability issues with mRNA vaccines cannot be ignored, as the mRNA’s tendency to break and the resulting loss of integrity are major concerns. For example, Moderna’s mRNA-1273 vaccine can be stored for six months at −20 °C, 30 days at 2–8 °C, but only 12 h at room temperature [168]. The short storage life of currently available mRNA vaccines limits their use in some settings. Therefore, improving mRNA integrity and stability is an important direction for enhancing mRNA vaccine quality.

#### Nucleotide modification

Nucleotide modifications have significant, multifaceted effects on mRNA quality. First, modifications can markedly reduce immunogenicity by preventing the mRNA from being recognized and degraded by the innate immune system in cells. For instance, modified nucleotides such as pseudouridine (Ψ) and N1-methylpseudouridine (m1Ψ) interfere with recognition by pattern recognition receptors (TLRs), protein kinase R (PKR), and others, thus reducing immune responses [49]. Second, modifications can improve mRNA stability and translation efficiency. For example, modifications such as m1Ψ and 5-methoxyuridine (5moU) not only decrease immunogenicity but also significantly enhance translation [169]. Preclinical studies have shown that pseudouridine-modified mRNA exhibits up to a tenfold increase in translation in various mammalian cell lines compared to unmodified mRNA and demonstrates enhanced translation persistence in mice [50]; similarly, modified mRNA can efficiently reprogram human cells into pluripotent stem cells, with the optimal effect achieved by co-modification with m5C and pseudouridine, while unmodified mRNA is too cytotoxic for reprogramming [170]. Subsequent studies revealed that m1Ψ-modified mRNA has better antigen expression and lower cytotoxicity than pseudouridine-modified mRNA [52]. In addition, nucleotide modifications can optimize mRNA structure for better in vivo functionality; for example, m6A modification can regulate mRNA splicing, transport, and translation [171].

CureVac’s CVnCoV vaccine, which did not use modified nucleotides, showed only 48.2% efficacy in COVID-19 prevention, while N1-methylpseudouridine-modified mRNA vaccines from Pfizer (BNT162b2) and Moderna (mRNA-1273) achieved over 90% efficacy. It indicates that N1-methylpseudouridine (m1Ψ) modification may impact translation accuracy. They analyzed the effects of 5-methoxyuracil (5-methoxyU), 5-methylcytosine (5-methylC), and m1Ψ modification on the translation process through in vitro translation experiments. It was found that m1Ψ-modified mRNA exhibited slower translation rates compared to unmodified mRNA, 5-methylcytosine (5-methylC), and m1Ψ modification on the translation process. They found that m1Ψ-modified mRNA translated at a slower rate than unmodified mRNA and produced a higher proportion of prematurely terminated peptide products. More importantly, m1Ψ modification may induce ribosomal frameshifting, thereby impairing translation fidelity. Further studies confirmed that recipients of the BNT162b2 vaccine (which underwent m1Ψ modification) developed an off-target cellular immune response to the predicted + 1 frameshift protein product, whereas recipients of the ChAdOx1 nCoV-19 vaccine (which did not utilize modified nucleotides) did not exhibit a corresponding reaction [172]. Although no adverse reactions caused by the + 1 frameshift protein product were identified in this study, and the safety of mRNA vaccines has been validated by extensive vaccination data, the potential impact of m1Ψ modification or other modifications on the protein translation process remains significant and should not be overlooked. This study indicates that mRNA sequence design is critical to avoid the + 1 frameshift. We should warn the mRNA research community to carefully investigate how the sequence design is correlated with + 1 frameshift.

#### Capping efficiency

Capping efficiency is a core parameter of mRNA quality, directly affecting its stability and translation efficiency. High capping efficiency (>95%) protects mRNA from degradation by 5’→3’ exonucleases (such as Xrn1) and extends its half-life. In addition, the cap structure binds to eukaryotic initiation factor 4E (eIF4E), enhancing ribosome recognition and loading efficiency, resulting in a 3- to 5-fold increase in protein expression. Uncapped or poorly capped mRNA (< 80% capping efficiency) exposes the 5’ triphosphate end, which can activate innate immune receptors such as RIG-I, induce interferon responses, and inhibit translation [173].

The capping method (e.g., co-transcriptional capping vs. enzymatic capping) directly affects capping efficiency. For example, the CleanCap^®^ technology can achieve >99% Cap1 capping efficiency, whereas traditional co-transcriptional methods achieve only about 70–85% and require rigorous quality control via HPLC or mass spectrometry [174].

#### Poly(A) tail

The poly(A) tail is a key regulatory element affecting mRNA quality, influencing both stability and translation efficiency. Addition of the poly(A) tail reduces the proportion of uridine bases in mRNA, thereby decreasing its recognition as a foreign nucleic acid by the immune system [44]. The poly(A) tail binds poly(A)-binding protein (PABP), forming a ribonucleoprotein complex that protects mRNA from 3’→5’ exonuclease degradation (such as by the CCR4-NOT complex) and extends its half-life. PABP works together with the 5’ cap to facilitate mRNA circularization, enhance ribosome recycling efficiency, and increase protein yields by 5–10 fold [40]. In addition, overly short poly(A) tails (< 30 nt) lead to insufficient stability, while excessively long tails (>200 nt) may induce immunogenicity or interfere with nucleocytoplasmic transport; in vaccine design, 100–120 nt poly(A) tails combined with optimized UTRs are often used to maximize expression [46]. The poly(A) tail is susceptible to deadenylase degradation; introducing spacer sequences (such as G/C bases or mixed nucleotides) can slow degradation [150]. For example, a team from HKUST replaced the terminal adenine in the poly(A) tail with cytosine, resulting in a 3–10 fold improvement in expression [175].

### Impurity control

In vitro transcription (IVT) is the core step in mRNA production. An optimized IVT system can reduce raw material use, lower production costs, yield high quantities and quality of mRNA, maintain physiological activity, and simplify downstream purification. However, IVT reactions readily produce a variety of contaminants, such as dsRNA, residual DNA templates, NTPs, DNA: RNA hybrids, cap analog residues, and non-nucleotide impurities, among which dsRNA, as a key impurity, has been a major focus.

#### Impact of DsRNA residuals

Double-stranded RNA (dsRNA), as an important pathogen-associated molecular pattern (PAMP), plays a central role in the host innate immune system. It triggers the release of interferons (IFNs) and a series of inflammatory cytokines by activating multiple pattern recognition receptors (PRRs), such as Toll-like receptor 3 (TLR3), protein kinase R (PKR), and RIG-I-like receptors (RLRs), thereby inducing a robust innate immune response [176]. This “double-edged sword” effect of immune activation is particularly significant in mRNA therapeutics. On one hand, structurally defined short dsRNA can serve as an effective immune adjuvant. For example, research by Tockary TA demonstrated that covalently linking short dsRNA to antigen-encoding mRNA strands can target RIG-I to achieve precise immune activation; quantitative control (1–5 “teeth”) enables precise regulation of immune stimulation intensity and effectively enhances the expression of dendritic cell (DC) surface markers (CD86, MHC I), thereby promoting antigen presentation capacity [177]. This strategy suggests the potential application of dsRNA in optimizing specific immunogenicity.

However, during in vitro transcription (IVT) processes, the procedure inevitably generates highly heterogeneous dsRNA byproducts. These dsRNA byproducts are characterized by random lengths (from < 40 bp to >1 kb), disordered sequences, and diverse conformations (such as hairpin structures and fully complementary double strands). This type of non-specific dsRNA can be recognized by various PRRs (such as MDA5 and TLR3), subsequently triggering excessive or unnecessary immune responses. This uncontrolled immune activation may not only lead to local inflammation (such as redness and swelling at injection sites) or systemic side effects (such as fever), thereby severely limiting the clinical dosage of mRNA vaccines, but may also cause significant adverse effects on their overall quality and therapeutic potential [178].

In-depth investigation of dsRNA-induced receptor activation mechanisms reveals their complexity and high specificity. For instance, PKR activation shows sensitivity to dsRNA length, typically requiring fragments longer than 30 bp for activation [179]; while TLR7/8 can be activated by dsRNA as short as 19–21 bp [180]. Furthermore, OAS1 activation depends on specific sequence motifs (such as WWN9WG) [180], but the sequence preferences and activation mechanisms of other OAS family members (such as OAS3) remain unclear. Nucleoside modifications, such as pseudouridine (Ψ) and N6-methyladenosine (m6A), although effective in reducing dsRNA generation and weakening immunogenicity [59, 181], carry potential risks that cannot be overlooked. For example, N1-methylpseudouridine (m1Ψ) has been reported to potentially cause ribosomal frameshifting mutations, thereby increasing translational toxicity [181]. In terms of immune balance, moderate levels of type I interferon (IFN-I) can enhance antigen presentation, but excessive activation may suppress target protein expression [182, 183]. Notably, even as low as 0.1% dsRNA concentrations can induce significant IL-6 production [183], highlighting the urgency of strictly defining safety thresholds for dsRNA.

#### Impact of RNA: DNA

The potential safety impact of RNA: DNA hybrids primarily involve triggering unintended innate immune responses. RNA: DNA hybrids, as novel pathogen-associated molecular patterns (PAMPs), can be accurately or erroneously recognized by various pattern recognition receptors (PRRs) within cells: In the cytoplasm, cGAS can bind these hybrids, catalyzing the generation of cyclic guanosine monophosphate-adenosine monophosphate (cGAMP), activating the STING–TBK1–IRF3 axis, and driving massive expression of type I interferons (IFN-α/β) and pro-inflammatory factors (such as IL-6, TNF-α, IP-10) [92]; Within endosomes, hybrids can serve as TLR9 ligands, activating dendritic cells (DCs), B cells, and macrophages through MyD88-dependent signaling cascades, accelerating the secretion of inflammatory factors such as IL-6 and TNF-α [91]; Additionally, they can activate the NLRP3 inflammasome, promoting the maturation and secretion of pro–IL-1β and pro–IL‑18 [93]. These signaling pathways can serve as adjuvants to enhance immunogenicity in vaccination, but if the response is excessive, it may lead to “cytokine storm,” triggering systemic inflammatory responses or even hemolytic uremic syndrome-like pathological changes. More seriously, if hybrids are misidentified by the immune system as persistent viral genetic material, they may break immune tolerance and induce systemic lupus erythematosus and Aicardi–Goutières syndrome (AGS). For example, hybrid accumulation caused by RNase H2 deficiency is considered a direct pathogenic mechanism of AGS [93]. In mRNA vaccines or viral vector systems, if large amounts of hybrids remain from the production process or form within the host (such as due to unexpected reverse transcriptase activity), they may also activate antigen-independent immune pathways, thereby weakening antigen-specific immune response effectiveness [91–93].

Furthermore, the presence of RNA: DNA hybrids can physically impede DNA replication fork progression. This replication fork stalling triggers DNA damage and may potentially lead to DNA strand breaks [184, 185]. Endogenous RNA: DNA hybrids exacerbate conflicts between replication forks and transcription, leading to replication stress and genomic instability, which are hallmarks of cancer and neurodegenerative diseases [184]. The entry of exogenous DNA-RNA hybrid chains into the cell nucleus is a complex process regulated by multiple factors, depending on the participation of key components such as nuclear pore complexes, nuclear transport receptors, and RanGTPase. Although the risk of gene integration is not high, potential risks may still exist. In summary, the presence of RNA: DNA hybrids increase the potential immunogenicity and gene integration risks of mRNA products, thereby affecting vaccine safety. Therefore, it is necessary to more effectively control RNA: DNA hybrids in mRNA vaccine production through methods such as optimizing in vitro transcription reaction conditions, improving DNA template quality, adding deoxyribonuclease (DNase1) for enzymatic digestion, and efficient purification [58, 59].

#### Strategies to control safety risks during the IVT process

To reduce the immunogenicity of in vitro synthesized mRNA, scientists have conducted extensive research, among which one of the most important findings is the use of modified nucleotides for mRNA synthesis, which can suppress innate immune activation and improve protein expression. Katalin Karikó and Drew Weissman discovered that with nucleotide modifications such as pseudouridine (Ψ), 5-methylcytidine (m5C), N6-methyladenosine (m6A), 5-methyluridine (m5U), or 2-thiouridine (s2U), cellular inflammatory responses were almost eliminated [49]. Their further studies confirmed that base modifications can reduce the innate immune effects of IVT mRNA on cells. Subsequent researchers have also found that RNA modified with pseudouridine, N6-methyladenosine, and 2-thiouridine has reduced ability to activate OAS and effectively delays RNase L-mediated degradation [181]; using pseudouridine, N1-methylpseudouridine (m1Ψ), and 5-methylcytidine reduces dsRNA production [64, 83], resulting in reduced immune responses; 5-methyluridine and pseudouridine not only significantly reduce mRNA immunogenicity but also greatly enhance translation efficiency.

In addition to base modification during the IVT process, various other strategies can address mRNA immunogenicity. T7 RNA polymerase (T7 RNAP) is highly promoter-specific and catalyzes RNA synthesis in the 5’→3’ direction but can also generate oligonucleotide and 3’-extended byproducts. Thus, optimizing T7 RNAP maintain high-efficiency transcription while reducing heterogeneity of RNA products is necessary. Modifications to T7 RNAP mainly include altering specific amino acid sites to improve the thermostability or transcriptional activity of T7 RNAP mutants; increasing transcription temperatures to effectively reduce dsRNA generation; and targeted mutations of the T7 RNAP nucleotide sequence to enhance enzyme stability [186]. These approaches can yield high-temperature-resistant T7 RNAP mutants with high transcriptional efficiency and low dsRNA levels. Other IVT reaction components, apart from T7 enzyme, NTPs, and DNA template, are also key limiting factors: lowering Mg^2+^ concentration reduces dsRNA [60, 187]; temperature affects enzyme-template binding, with optimal temperatures reducing dsRNA formation; addition of denaturants (urea or formamide) weakens adverse interactions between poly(A) tails and products, prevents reverse transcription, and can lower dsRNA content by about 60–70% [188].

Reducing byproduct formation during IVT is best complemented by post-synthetic purification strategies. The 5’ cap (m7G) and 3’ poly(A) tail are characteristic features of mRNA and provide selection tags for extraction and purification, leading to the development of various purification methods—mainly lithium chloride (LiCl) precipitation, magnetic bead purification, and chromatographic techniques. The LiCl method leverages Li^+^ to minimize electrostatic repulsion between molecules at specific pH, precipitating RNA for subsequent extraction by centrifugation, although it is ineffective for small RNA and residual Li^+^ inhibits mRNA activity [189]. Magnetic bead purification uses modified iron oxide beads that bind mRNA and can be separated by magnetic fields; common bead surface modifications include hydroxyl, carboxyl, Oligo(dT), and streptavidin. Both methods are simple and yield highly pure mRNA but are only suitable for small-scale applications and not industrial production, where chromatographic purification is the mainstream approach due to scalability and controllability. Common methods include ion exchange, core chromatography, hydrophobic and affinity chromatography, with affinity chromatography (e.g., Oligo(dT) columns) widely used: “high-salt binding, low-salt elution.” However, this method cannot distinguish ssRNA from dsRNA with poly(A) tails, so additional purification (e.g., hydrophobic or anion exchange chromatography) may be required. Ceramic hydroxyapatite (CHT) chromatography and cellulose chromatography can also be used for mRNA purification: CHT exploits the negative charge of nucleic acid phosphate backbones binding to Ca^2+^ on CHT, with stepwise increases of phosphate to allow phosphate in the buffer to compete and separate dsRNA from ssRNA [81]; BioNTech demonstrated that dsRNA selectively binds cellulose in ethanol buffers, allowing the separation of dsRNA from ssRNA regardless of mRNA length, sequence, or nucleotide composition, at least 90% of dsRNA can be removed [64]. In practice, combining multiple purification strategies may best match quality standards and minimize the impact of IVT byproducts on mRNA purity.

In summary, mRNA preparation must be strictly controlled at the source—rigorously standardizing each step in the IVT process, minimizing byproduct generation, and establishing a robust quality management system to ensure the stability and safety of mRNA vaccines.

### Stability

During storage and transportation, mRNA vaccines must maintain their physical stability. Appropriate storage conditions and packaging materials can prevent mRNA degradation and denaturation, ensuring good immunogenicity at the time of use. Temperature is a key factor affecting mRNA vaccine stability. Studies have shown significant differences in mRNA vaccine stability at different temperatures. As storage temperature increases, mRNA integrity gradually declines, leading to reduced vaccine potency. Repeated freeze-thaw cycles may also cause changes in the physical and chemical properties of mRNA vaccines, thereby affecting their stability. Additionally, physical shocks and pH fluctuations can further impact vaccine stability [168].

Under stress environments such as frozen storage, mRNA-LNPs may encounter challenges including mechanical stress from ice crystals, pH fluctuations, and regional variations in internal ionic strength [190, 191]. For example, storage at −80 °C without cryoprotectants leads to decreased protein expression, which is associated with nanoparticle aggregation; external factors such as vibration and light exposure can also induce similar structural changes, suggesting the need for strict aggregation risk control during R&D and clinical phases [192]. Studies on freeze-thaw stability have found that LNPs with sucrose cryoprotectant still aggregate easily at −80 °C, while storage at 4 °C, −20 °C, and − 200 °C can maintain a particle size distribution similar to freshly prepared particles. This may be due to the intermediate cooling rate at −80 °C producing a mixed ice/glass state prone to aggregation [193]. Moderna’s cryo-EM studies have visually demonstrated changes in LNP particle size and morphology under physical stress, showing aggregation and mRNA leakage after freeze-thaw cycles [194]. Pfizer’s research indicates that during storage at −20 °C, PEGylated lipid conformational changes and lipid rearrangement occur, with cryo-EM showing increased vesicle formation; this structural change is attributed to the pH drop upon freezing, which may affect LNP stability and biological activity [195]. Additionally, available data show that Moderna’s vaccine can be stably stored for 6 months at −20 °C, and Pfizer-BioNTech’s vaccine for 6 months at −80 to −60 °C. However, when stored at 2–8 °C, the shelf lives of Moderna and Pfizer-BioNTech vaccines are rapidly shortened to 30 days and 5 days, respectively [168].

### Critical role of the LNP delivery system

Messenger RNA (mRNA) delivery has become an innovative modality for the prevention and treatment of various diseases (such as cancer, infections, and genetic disorders). The development of efficient delivery methods has addressed the problems of poor delivery efficiency and immunogenic responses associated with naked nucleic acid cargoes [196]. Delivery carriers can be classified as biological (mainly viral) or non-biological (e.g., lipid and peptide) categories. Viral vectors have received clinical approval, but their use is highly limited due to risks such as insertional mutagenesis, small payload capacity, anti-vector immunogenicity, and safety issues. Non-viral carriers include polymers and lipid nanoparticles (LNPs). As early as the 1970 s, polyacrylamide polymer nanoparticles were applied for the encapsulation and delivery of non-diffusible compounds; compared to treatment with free fluorescein, the accumulation of fluorescein molecules in cultured fibroblasts was significantly enhanced [197]. Currently, lipid nanoparticle (LNP) systems are the main non-viral gene drug delivery systems, and as a delivery platform, LNPs have demonstrated robust capabilities.

Liposomes represent the first generation of LNPs, and in 1995, Doxil™ (liposomal doxorubicin) successfully entered the clinic as a treatment for ovarian cancer and is one of the earliest marketed examples of LNP technology [198]. The first approved drug to use nucleic acid encapsulated in LNPs was Onpattro™, which received regulatory approval in August 2018. This LNP formulation delivers small interfering RNA (siRNA) to inhibit the synthesis of transthyretin (TTR) protein in the liver for the treatment of hereditary transthyretin amyloidosis-induced polyneuropathy [198]. Although LNPs have been extensively studied over the past twenty years, their widespread use in mRNA delivery did not occur until the 2019 COVID-19 pandemic, when LNP-mRNA vaccines were administered to large populations.

#### The impact of lipids on delivery efficiency and tissue distribution

LNPs are mainly composed of ionizable lipids, helper phospholipids, cholesterol, and polyethylene glycol lipids (PEG-lipids). Ionizable lipids promote spontaneous assembly of mRNA into virus-sized particles and facilitate mRNA release in the cytoplasm. PEG prolongs the complex’s half-life. Cholesterol acts as a stabilizer, increasing complex stability, and phospholipids support the formation of the lipid bilayer structure. Adjusting the proportions of these four components allows fine-tuning of LNP size [199].

Ionizable lipids, as the core component of LNPs (typically accounting for a 50% molar ratio), play a significant role in controlling delivery efficiency and tissue distribution. The pKa value of ionizable lipids, which is a key molecular feature of amino lipids, determines their ability to mediate effective hepatic gene silencing in vivo. Studies on siRNA delivery efficiency and the pKa of ionizable lipids show an optimal range between 6.2 and 6.5, confirming that DLin-MC3-DMA is an excellent lipid for siRNA delivery [200]. The linker between the ionizable headgroup and the hydrocarbon tail can also modulate tissue targeting ester linkers favor liver delivery, while amide linkers tend to target the lungs [201]. Ionizable lipids usually contain 1–4 hydrophobic tails with 8–20 carbon atoms, which can be saturated or unsaturated, straight or branched. More branches introduce a more conical structure to the ionizable lipid and enable stronger membrane-disrupting capability when paired with anionic phospholipids in endosomes; SM-102 and ALC-0315 are examples of branched-tail lipids [202]. The alkyl chain length of the tails greatly impacts LNP organ distribution: shorter tails favor hepatic mRNA expression, while longer tails may steer LNPs towards the spleen [203]. Structure-activity relationship studies have shown that the alkyl chain length in the branched tails of cationic lipids substantially affects efficacy and organ selectivity: chains of 8–10 carbons on the amine side mediate high in vivo mRNA expression, whereas the alkyl length adjacent to phosphate groups affects organ preference—shorter chains (9–12 carbons) support hepatic mRNA expression, while longer chains (13–16 carbons) shift protein expression to the spleen [120].

The proportions of lipid components in LNPs significantly influence their tissue distribution and pharmacokinetics in mice and other animals. Helper lipids typically represent 10% molar ratio and also affect tissue targeting. With the commonly used Onpattro formulation (ionizable lipid/helper lipid/cholesterol/PEG-lipid at a molar ratio of 50/10/38.5/1.5), about 90% of injected LNP-mRNA is transferred to and cleared by the liver within 30 min after intravenous administration. Addressing this, Pieter Cullis’s team found that increasing helper lipid content gradually shifts the LNP-mRNA system from individual solid core structures to a “bleb” morphology—dual-chamber, vesicle-like structures surrounding a smaller solid core that offer a longer circulation lifetime and significantly increase LNP-mRNA accumulation in extrahepatic tissues [136]. Helper lipids play a role in encapsulating and stabilizing LNPs, and influence their delivery efficiency and in vivo distribution. Studies have found that formulations containing DOPE preferentially accumulate in the liver, while LNPs substituted with DSPC instead of DOPE accumulate in the spleen [204]. Cholesterol plays a role in regulating the membrane fluidity of LNPs and stabilizing their structure, aiding in nucleic acid encapsulation and enhancing encapsulation efficiency. Research has revealed that cholesterol can also promote membrane fusion and participate in the endosomal escape process. Cholesterol interacts with apolipoprotein E (ApoE), causing redistribution of LNP components—a process critical for endosomal escape—thereby influencing payload release [102]. The structure of PEGylated lipids (such as PEG length, degradable linkers, terminal groups, etc.) can regulate immunogenicity and safety, making them a key component in delivery systems such as mRNA-LNP [132]. The preparation method of LNPs also influences their quality attributes, transfection efficiency, and in vivo distribution. For instance, studies have shown that performing ultrafiltration in two steps during LNP preparation—removing ethanol and adjusting pH separately—affects the particle fusion process, which results in larger LNP particle sizes, fewer empty LNPs, and more uniform morphology [205].

#### Impact of LNP structure on delivery efficiency and tissue distribution

The particle size of lipid nanoparticles (LNPs) is one of the key parameters influencing their cellular uptake efficiency, in vivo distribution, and delivery outcomes. In terms of circulation time and targeting, small particles (~ 50–100 nm) have a longer circulation half-life since they are less likely to be cleared by the mononuclear phagocyte system (MPS), though they may be nonspecifically taken up by liver Kupffer cells [206]. Larger particles (>200 nm) are more readily cleared by the MPS, resulting in reduced bioavailability, but may passively accumulate in tumor tissues due to the enhanced permeability and retention (EPR) effect [207]. Regarding endocytic pathways, small particles (~ 50–100 nm) are more efficiently internalized via clathrin-mediated endocytosis (CME), which is the main pathway for nanoparticle uptake in most cell types [208]. The high-curvature surfaces of smaller particles facilitate clathrin binding and enhance vesicle formation efficiency. Larger particles (>100 nm) tend to enter cells via macropinocytosis or caveolae-mediated endocytosis; these pathways are generally less efficient and may result in nanoparticle entrapment within endosomal vesicles, reducing effective cargo release [209].

The internal structure of LNPs also influences their tissue distribution and cellular uptake. Studies investigating how LNP buffer exchange variables (ethanol removal, pH increase, ionic strength increase) affect internal structure and transfection efficiency showed that, in “first generation” lipid (e.g., KC2)-based mRNA-LNP systems, inducing bleb structures with high ionic strength could achieve higher in vivo transfection efficiency and greater splenic distribution than ALC-0315 or SM-102-based systems [210].

It is worth mentioning that most studies on LNP biodistribution are based on in vitro experiments or small animal models such as mice; research in primates and large animals is relatively scarce. Since the size of the circulatory and lymphatic systems varies with animal size, uptake and metabolic kinetics can differ, so more data are needed on LNP biodistribution in humans.

#### Relationship between LNP particle size and immune targeting

The size and distribution of LNPs have important effects on their in vivo behavior and performance. LNP diameters typically range from several dozen to several hundred nanometers, and their size is crucial for overall drug targeting and circulation time. After a vaccine enters the human body, antigens are recognized by pattern recognition receptors (PRRs) on antigen-presenting cells (APCs) such as macrophages and dendritic cells (DCs), and transported to draining lymph nodes or the spleen. Depending on the antigen, B cells may recognize it directly, or it may be presented to T cells in immune organs, thereby generating antigen-specific T cells or reactivating dormant/inactive T cells, ultimately eliciting new immune responses or enhancing existing ones [211].

**Fig. 12:**
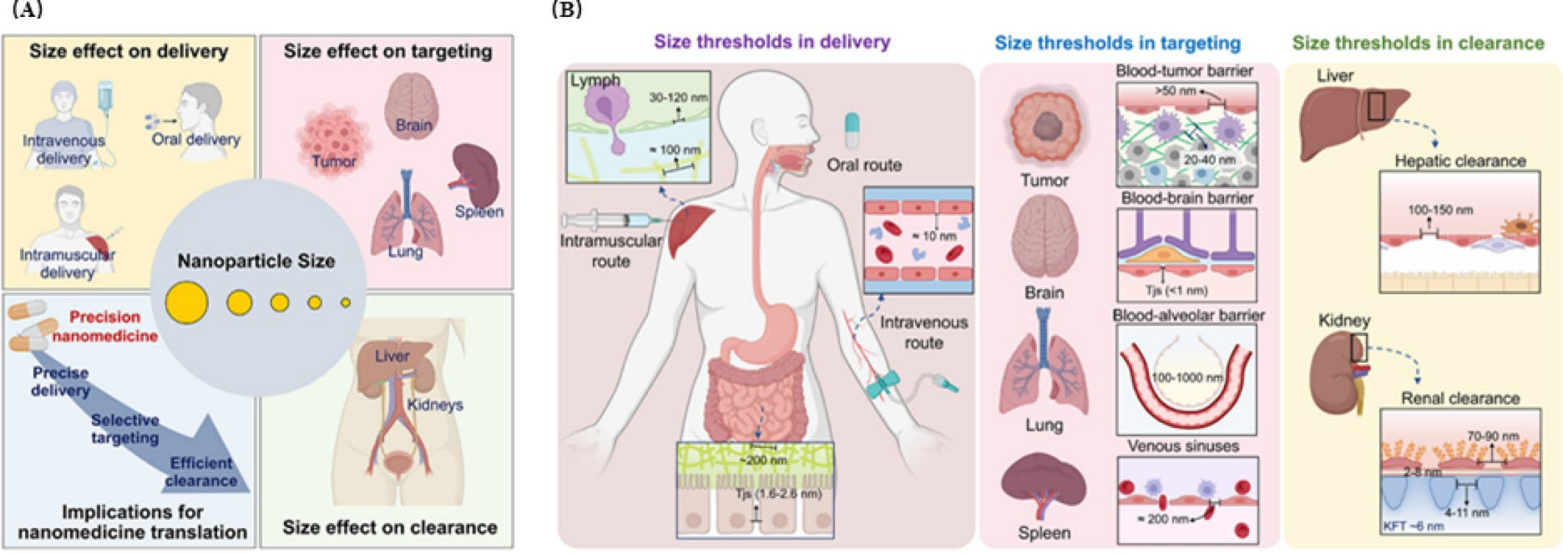
(**A**) Effect of nanoparticle size on in vivo transport; (**B**) Size Thresholds in the Body

Nanoparticle size critically determines in vivo translocation because size thresholds differ in organs and tissues: normal capillary endothelium has ~ 10 nm junctions, tumor vasculature typically expands to ~ 50 nm, liver vasculature has fenestrations of 100–150 nm, kidney glomerular filtration threshold is ~ 6 nm, while mucus in the intestine has a porous structure (~ 200 nm) and tight epithelium (1.6–2.6 nm). These size barriers dictate different biodistribution behaviors *in viv*o [212] (Fig. 12). Studies show that particles 20–200 nm in size are more readily taken up by APCs such as DCs and macrophages. For example, LNPs around 100 nm are efficiently internalized into DCs via clathrin-mediated endocytosis, while particles >500 nm may rely on phagocytosis, which is less efficient [213]. The DC uptake rate for 50 nm LNPs is three times higher than for 200 nm, directly influencing antigen presentation strength [214].

Passive targeting based on LNP particle size and surface properties is determined by physical parameters [215]. Smaller particles are more likely to evade clearance by mononuclear phagocytes and have longer circulation half-lives. Small molecules (< 20 kDa) or nanoparticles (< 10 nm) are rapidly filtered by the kidney after injection due to its innate blood-filtering function [216, 217]. Larger molecules (>20 kDa) or nanoparticles (10–100 nm) can passively diffuse into lymphatic vessels through inter-endothelial junctions. Research indicates that the optimal particle size for direct lymph node delivery is 20–50 nm [218]. LNPs of 50–100 nm preferentially enters the lymphatic system through clathrin- or caveolae-mediated pathways and can be captured by lymph node DCs. Particles >100 nm are more likely to be trapped in the interstitial space and need to be taken up by APCs before reaching the lymph nodes. For example, Moderna’s approved mRNA-1273 COVID-19 vaccine utilizes LNPs sized 80–100 nm, achieving a balance between lymphatic trafficking (peak lymph node concentration within 48 h) and APC activation efficiency [219]. Mark E. Davis et al. reported that nanoparticles of 75 ± 25 nm preferentially target the renal mesangium [219].

In summary, LNP particle size requires precise tuning according to application scenarios. Reducing size by 10 nm increases specific surface area by approximately 15%, which significantly changes surface ligand density and the protein corona. Therefore, optimal particle size parameters for each application must be experimentally determined.

#### Impact of ionizable lipids and pKa value on LNP delivery systems

Among the key components of LNPs, ionizable lipids play a crucial role in encapsulating, protecting, and transfecting mRNA cargo into cells. In an acidic environment, ionizable lipids carry a positive charge: during LNP assembly, the positively charged ionizable lipids facilitate interaction with negatively charged mRNA, tightly encapsulating mRNA inside the LNP. Additionally, they can disrupt cellular membranes, promoting endosomal escape after cellular internalization. At neutral pH, ionizable lipids remain neutrally charged, which helps minimize interaction with anionic cell membranes, reduce LNP toxicity and immunogenicity, and improve pharmacokinetic properties [220]. Ionizable lipid structure generally consists of a hydrophilic head group, two hydrophobic tails, and a linker connecting these domains [183]. Typically, amino/hydroxyl groups serve as hydrophilic heads, linoleyl or branched aliphatic chains are used as hydrophobic tails, and esters are frequently selected as linkers to achieve efficient mRNA delivery [221].

The negative surface charge and large size of mRNA hinder its cellular entry, and naked mRNA is highly susceptible to nuclease degradation and renal clearance. Exogenous RNA can also be detected by the innate immune system, triggering immune responses [222]. Therefore, the delivery system plays a crucial role in RNA therapeutics. Key attributes determining nanoparticle efficacy and safety include particle size, shape, surface charge, surface area, and ionization constant (pKa) [223–225]. Efficient cytoplasmic delivery of RNA requires endosomal escape facilitated by ionizable nanoparticles [226]; thus, optimizing these characteristics is essential for developing successful mRNA-LNP formulations.

The dissociation constant (pKa) determines the ionization behavior and surface charge of nanoparticles, thereby affecting their stability, efficacy, and toxicity. Surface charge also influences cellular uptake, endosomal release, and biodistribution [227, 228]. The apparent pKa of a nanoparticle reflects the average ratio of all ionizable and deprotonated groups on the particle and is not the intrinsic pKa value of a single molecule; typically, a nanoparticle’s apparent pKa is lower than that of its individual components [229]. As pH decreases, ionizable amine groups on nanoparticles transition from deprotonated to protonated states. By tuning the apparent pKa in LNP-siRNA formulations, cell-specific activity can be achieved [230]. Studies indicate that gene silencing activity correlates more closely with pKa than with particle size or siRNA encapsulation efficiency. LNPs with a pKa between 6 and 7 show good gene silencing, whereas those with pKa between 3 and 6 exhibit poor stability and cellular uptake, resulting in lower silencing efficiency [231]. Optimized LNPs with a pKa of 6.2–6.5 are effective for hepatic siRNA delivery [200]. Additionally, structural variations affect both physicochemical parameters and in vivo activity. Nanoparticles with a pKa of 6–6.6 and calculated lipophilicity of 10–14 demonstrate good in vivo efficacy [232].

To develop an efficient mRNA vaccine delivery system, Moderna synthesized 30 lipid candidates, finding that LNP pKa is a key determinant of immunogenicity. The optimal pKa range for intramuscular delivery was 6.6–6.9; SM-102 (pKa 6.68) was ultimately selected for the COVID-19 vaccine mRNA-1273 due to its favorable biodegradability, tolerability, and immunogenicity [116]. Moreover, LNPs initially designed for siRNA can be modified to deliver mRNA. For example, C12-200 LNPs, originally for siRNA, were optimized by altering lipid ratios and structures, resulting in a 7-fold increase in mRNA expression; the apparent pKa of the improved LNP shifted from 7.25 to 6.96. Interestingly, the two LNPs showed similar siRNA delivery efficacy, highlighting differences in optimal parameters for siRNA and mRNA formulations [233].

Current research demonstrates that nanoparticle structure and physicochemical properties are vital for RNA delivery. Although endosomal escape mechanisms of LNP vary, across cell types and require further investigation, the current hypothesis involves three steps: a) the acidization of ionizable lipid in endosome and the ionized lipid can form an ion pair with endosomal membrane anionic lipids such as phosphatidylserine; b) the ion pair therefore adopts a molecular ‘cone’ shape and converts lipids from lamellar phase to hexagonal phase; c) Hexagonal phases do not support bilayer structure and are associated with membrane fusion and membrane disruption [129]. In contrast, apparent pKa is a reliable indicator for predicting the RNA-encapsulation efficiency of nanoparticles, with strong correlation to efficacy and toxicity; particles with optimal apparent pKa show effective endosomal escape and therapeutic results. In conclusion, using apparent pKa as a design standard aids in identifying effective and safe RNA-based therapeutics. The ideal pKa depends on many factors—carrier structure, target tissue, and delivery route—making it difficult to recommend one universal value for all applications, but a pKa of 6–7 is considered optimal for nanoparticles intended for RNA therapeutics.

#### Safety risks of LNP delivery systems

LNPs can activate the innate immune system and promote the generation of adaptive immune responses due to their unique physicochemical properties and compositional components. As mentioned previously, they can mediate innate immune responses through activation of Toll-like receptors (TLRs), which recognize specific molecular patterns on LNPs, thereby activating downstream signaling pathways and leading to the release of inflammatory cytokines. They can also activate the type I interferon pathway, promoting the production of type I interferons. Type I interferons play important roles in antiviral immunity, but excessive type I interferon responses may also lead to inflammation and immunopathology.

Recent research indicates that endosomal damage is one of the primary causes of LNP-induced inflammatory responses. LNPs primarily enter cells through endocytosis. To release mRNA into the cytoplasm for protein translation, LNPs need to escape from endosomes. However, this escape process is often accompanied by damage to the endosomal membrane. Endosomal membrane damage leads to the formation of pores in the membrane, which are recognized by damage sensors in the cytoplasm, subsequently activating inflammatory responses. Galectins are one important class of damage sensors that can recognize damage on endosomal membranes and recruit downstream inflammatory signaling molecules, triggering inflammatory responses [234].

Ionizable lipids are key components in LNPs, affecting nucleic acid encapsulation, cellular uptake, and endosomal escape. Different ionizable lipids possess different physicochemical properties, such as pKa values, hydrophobicity, and charge distribution. These properties all influence the interactions between LNPs and cell membranes, endosome formation, and the manner of LNP release from endosomes. For example, certain ionizable lipids may possess properties that disrupt endosomal membranes, leading to the formation of larger pores in endosomal membranes. Conversely, other ionizable lipids may be able to stabilize endosomal membranes, reducing pore formation. The size and number of pores in endosomal membranes are direct causes of LNP-induced inflammatory responses [235]. Selecting biodegradable lipids can accelerate LNP degradation and clearance in vivo, thereby reducing their accumulation in the body and weakening inflammatory responses.

Helper lipids play roles in stabilizing structure, regulating biodistribution, and affecting intracellular transport in LNPs. Research by Muattaz Hussain et al. demonstrates that the types and proportions of helper lipids such as phospholipids and sterols also affect LNP immunogenicity. Therefore, by selecting appropriate helper lipids, the interactions between LNPs and the immune system can be modulated, thereby reducing inflammatory responses. This series of studies also indicates that rational LNP design must consider the complete lipid composition, not just ionizable lipids, to more effectively optimize LNP design for specific therapeutic needs [235].

The impact of LNP-induced inflammatory responses on vaccine efficacy is bidirectional. Moderate inflammatory responses can enhance vaccine immune responses, while excessive inflammatory responses may lead to immunosuppression and adverse reactions. Therefore, it is necessary to carefully evaluate the inflammatory characteristics of LNPs and take measures to optimize LNP design and composition to reduce inflammatory responses and improve vaccine effectiveness and safety.

### Methods for controlling critical quality attributes (CQAs)

The critical quality attributes (CQAs) of mRNA vaccines span multiple production stages and have a major impact on immunogenic outcomes. Therefore, accurately measuring and reflecting CQA values and changes is crucial for vaccine quality control.

#### mRNA integrity detection methods

For the study of mRNA integrity, gel electrophoresis is one of the most common techniques, including native agarose gel electrophoresis and formaldehyde-denatured agarose gel electrophoresis. mRNA molecules are negatively charged and migrate from the negative to the positive electrode under an electric field; the pore size of the agarose gel allows different lengths of mRNA molecules to be separated based on their migration rates. However, traditional methods like this, while low-cost, have poor quantification, usually relying on imaging of nucleic acid bands and subsequent densitometric analysis, resulting in inaccurate quantification, poor reproducibility, and lack of scalability for automated, high-throughput analyses.

In contrast, capillary electrophoresis enables real-time monitoring of fluorescence intensity during sample separation, digitized recording of signal strength, and automated instrumental analysis. Currently, capillary electrophoresis can analyze up to 96 samples in one run, with high sensitivity and resolution [236].

Recently, ion-pair reversed-phase liquid chromatography (IP-RP) has also been used for analyzing mRNA fragment impurities. AstraZeneca reported an analytical method capable of efficiently separating nucleic acid molecules within 1000 nt for analyzing mRNA integrity under various stability conditions [237]. Tokyo Metropolitan University developed an RP-HPLC method for separating RNA fragments up to 5000 nt, potentially allowing analysis of full-length mRNA and fragment impurities [238]. BioNTech used IP-RP to study fragment impurities and functionality of the marketed COVID-19 vaccine BNT162b2.

#### Capping efficiency detection methods

For capping efficiency, RP-LC-MS is the USP-recommended method. The mRNA is digested near the 5’ end to generate fragments suitable for mass spectrometry. LC-MS not only identifies the presence of cap structures [239–241] but can also differentiate between cap0, cap1, and cap2; determine the proportions of cap0 and cap1 in a sample; and detect intermediates in the capping process. Novartis used LC-MS to monitor capping efficiency and successfully optimized the ARCA reaction by fine-tuning conditions [242]. Inagaki et al. analyzed a new cap analog with a PureCap tag using LC-MS and RP-HPLC, increasing the maximum capping efficiency from ~ 80–90% to 100% [243]. These studies confirm that LC-MS is an effective method for mRNA cap detection and quantification, supporting manufacturing process development and product quality control.

Nanopore sequencing is a new platform technology that enables direct sequencing of nucleic acid molecules (including mRNA) and their modifications [244–246]. A single RNA molecule is driven through a protein nanopore embedded in a membrane, and the sequence is determined by measuring current changes as the molecule passes through. Nanopore technology was initially used to detect modifications such as N6-methyladenosine (m6A) in RNA [247–249]. Recently, Wang et al. reported engineered nanopores that can directly identify a variety of RNA modifications, including N7-methylguanosine (m7G), pseudouridine (ψ), N6-methyladenosine (m6A), 5-methylcytidine (m5C), N1-methyladenosine (m1A), 5-hydroxymethylcytidine, N6,2′-O-dimethyladenosine, N4-acetylcytidine, and A-to-I editing [250]. Using machine-learning algorithms, detection accuracy can reach 0.996.

Compared with LC-MS, nanopore RNA sequencing offers a simpler sample preparation process and high-resolution functional distinction of RNA molecules and their modifications. However, the experimental process is complex and costly, and specialized data analysis is required, which currently limits the widespread adoption of nanopore sequencing as a routine RNA analysis method. Therefore, LC-MS remains the gold standard for mRNA cap structure analysis.

#### Poly(A) Tails detection methods

Currently, various methods have been developed for the detection of mRNA Poly(A) tails, including Northern blotting, RT-PCR, next-generation sequencing, RP-HPLC, and LC-MS. Traditional RNA blotting techniques, Northern blotting, and RT-PCR cannot provide precise information on Poly(A) tail length and composition. At present, LC-MS remains the mainstream method for Poly(A) tail analysis. Enzymatic digestion and dT magnetic bead enrichment can be used to isolate the Poly(A) tail from mRNA, followed by size exclusion chromatography to determine the average length, or high-resolution IP-RP-HPLC for detailed analysis of Poly(A) tail length and modifications. Gilar M et al. developed an ion-pair reversed-phase liquid chromatography (IP-RP-HPLC) method that can distinguish poly(A) tails differing by a single nucleotide [251]. Beverly M et al. developed an LC-MS method that provides tail-length information at single-nucleotide resolution [252]. The development of next-generation sequencing has made high-throughput, precise analysis of poly(A) tails possible. Eisen T J et al. used PAL-seq to dynamically display poly(A) length changes [253, 254]. Chang H et al. used TAIL-seq to detect base modifications (guanylation and uridylation) in the poly(A) tail, enabling measurement of poly(A) length and modification status [255]. However, both PAL-seq and TAIL-seq rely on PCR amplification of cDNA, and the existence of long polymers inevitably introduces amplification bias. The emergence of Nanopore DRS (Direct RNA Sequencing) technology has brought about a revolutionary breakthrough in RNA research. This DRS approach is free from cDNA synthesis and PCR amplification, thus enabling the direct, real-time sequencing of a single RNA molecule and generating full-length, strand-specific reads. Workman R E et al. characterized Poly(A) length and base modifications via nanopore sequencing [256], However, this DRS approach has certain limitations when applied to the quality control (QC) of poly(A) tail length and distribution in mRNA vaccines [257]. One key limitation is the error rate. Although nanopore sequencing can handle long RNA strands and directly sequence native RNA, it generally has a higher error rate compared to other high-throughput sequencing methods. It can affect the accuracy of poly(A) tail length measurements and may require additional bioinformatics tools to correct errors. ⁠ Another limitation is the potential for RNA secondary structures and modifications to interfere with the sequencing process. The presence of these high order structures can lead to stalling or misreading by the nanopore, which can complicate the accurate determination of poly(A) tail lengths. Additionally, the throughput of nanopore sequencing, while improving, may still be lower than that of short-read sequencing technologies, making it less efficient for large-scale QC processes. Furthermore, the requirement for specialized equipment and expertise in data analysis can pose a barrier to widespread adoption in QC workflows. The need for robust and standardized protocols, as well as the continuous development of more accurate and efficient basecalling algorithms, is crucial for overcoming these limitations and enhancing the utility of nanopore sequencing in mRNA vaccine quality control.

#### Modified nucleotides detection methods

For modified nucleotides, researchers have developed several quantitative methods for site and content detection to improve the quality control of modifications. For example, Qing Dai and colleagues developed BID-seq, which tests different bisulfite kits, reaction conditions, and reverse transcriptases, calibrates sequence-dependent deletion rates with internal probes of various Ψ levels, and calculates the stoichiometry of Ψ-modified sites; this enables quantification of pseudouridine location and abundance [258]. Zhang M. et al. developed the PRAISE method. Based on quadruplet nucleotide mapping, it enables comprehensive, transcriptome-wide quantitative analysis of Ψ modifications [259]. PRAISE uses selective chemical marking of Ψ via optimized bisulfite/sulfite ratios. During reverse transcription, marked Ψ sites generate nucleobase deletion signatures, which can be captured by high-throughput sequencing for single-base resolution detection of Ψ across the transcriptome [259]. Xu H. et al. developed the BACS method, utilizing 2-bromopropionamide-assisted cyclization sequencing, achieving quantification of pseudouridine (Ψ) to cytidine (C) transition with single-base resolution and precise localization of Ψ even in high-density modified regions and consecutive uridine sequences. This method can also detect A-to-I editing and N1-methyladenosine (m1A) [260].

#### DsRNA content detection method

Precise detection and standardized regulation of dsRNA represent critical challenges that urgently need to be addressed. Currently, ELISA is the preferred method for dsRNA quantitation, using specific antibodies for capture and analysis. Classic J2 and K2 antibodies (generated by immunizing mice) effectively recognize dsRNA [261]. Recent research shows that M2 and M5 antibodies have comparable detection capabilities and can detect dsRNA longer than 40 bp [262], whereas cannot effectively detect dsRNA fragments shorter than 40 bp [178, 263], which leading to underestimation of short-chain dsRNA residual risks. Meanwhile, quantitative methods such as reverse-phase high-performance liquid chromatography (RP-HPLC) can provide total dsRNA amounts but cannot distinguish their length or structure. These technical bottlenecks further result in inconsistent dsRNA safety threshold standards within the industry, with enormous differences in corporate limits (ranging from ≤ 0.1%, < 0.5% to ≤ 2000 pg/µg RNA) [264], and there is a general lack of supporting clinical-relevant data.

Other assays, such as dot blotting, can detect dsRNA but lack accurate quantification [265]. Acridine orange staining with gel electrophoresis is cumbersome and less accurate [61]. Furthermore, more new advanced analytical technologies and methods have also been developed for the sensitive and accurate detection of dsRNA. Homogeneous time-resolved fluorescence (HTRF) method is a luminescence-based assay that utilizes specific antibodies to detect dsRNA. It involves the use of a donor and an acceptor fluorophore, which interact through a FRET (Förster Resonance Energy Transfer) mechanism when in close proximity [266]. This FRET method possesses excellent advantages such as high specificity, sensitivity and accuracy, and also well suitable for high-throughput testing and provides quantitative results with a wide dynamic range. A microfluidic electrophoresis method has also been developed for the residual dsRNA impurities detection in mRNA vaccines and therapeutics [267]. This novel method combines enzymatic isolation of dsRNA with microfluidic electrophoresis for precise quantification. It offers a high-resolution approach to detect and quantify dsRNA impurities with high sensitivity and precision. Although this microfluidic electrophoresis method is suitable for detailed characterization of dsRNA impurities, but can be more complex and time-consuming compared to other methods. In summary, the choice of residual dsRNA analysis method in mRNA vaccines depends on the specific requirements of the study, including sensitivity, throughput, accuracy or more detailed characterization, such as dsRNA length. HTRF and ELISA offer high sensitivity and are suitable for high-throughput screening, while the dot blotting and gel electrophoresis methods are simpler but less sensitive. Enzymatic isolation combined with microfluidic electrophoresis provides a high-resolution and precise quantification but requires specialized equipment and expertise. In any case, there is an urgent need to establish length/structure-based dsRNA classification standards and develop highly sensitive detection methods capable of comprehensively characterizing dsRNA heterogeneity.

#### Other detection method

mRNA-LNP particle size and distribution can be monitored by dynamic light scattering (DLS), cryo-electron microscopy (Cryo-EM), and asymmetrical flow field-flow fractionation with multi-angle light scattering (FFF-MALS). DLS can directly measure particle size in samples in their undiluted state; it is simple, rapid, delivers stable results, and is suitable as a release test for critical quality parameter “particle size.” DLS is also the method recommended by the USP *Draft Guidance for Analytical Procedures for Quality Testing of mRNA Vaccines and Therapeutic Products*, but its accuracy can be affected by sample polydispersity. Therefore, as recommended in China’s *Technical Guidelines for Pharmaceutical Research of mRNA Vaccines for the Prevention of COVID-19 (Trial)*, appropriate structural confirmation methods such as cryo-EM or static light scattering should be used to confirm the suitability of DLS.

The surface charge of mRNA-LNP can be monitored by electrophoretic light scattering (ELS), TNS fluorescent dye binding, or capillary isoelectric focusing electrophoresis (cIEF). ELS can also directly determine charge without sample dilution; it is simple, rapid, gives stable results, and is suitable as a release test for critical parameter “zeta potential.” ELS is included as a recommended method in the *Draft Technical Guideline for Quality Control of Nanomedicine* in China, but for samples with low absolute Zeta potentials, the method’s sensitivity or accuracy may be insufficient.

Meanwhile, mRNA vaccine quality control methods continue to evolve. The 2024 USP (3rd edition) recommends several quality control methods for mRNA vaccines [268]. High-throughput sequencing and reverse transcription–digital PCR, introduced in this edition, reflect ongoing development in key quality attribute control for mRNA vaccines.

## Future challenges and opportunities

Despite rapid advances in the field, several practical and scientific hurdles remain before mRNA vaccines can achieve their full global impact. One of the most pressing issues is scalability of manufacturing. Current IVT and LNP platforms have proven highly adaptable for clinical development, but expanding to worldwide supply requires greater robustness in raw material sourcing, batch-to-batch enzyme consistency, and streamlined production pipelines. Integration of continuous manufacturing and in-line quality monitoring—such as real-time particle sizing or dsRNA quantification—could significantly improve reproducibility and reduce costs. Equally important, developing formulations with improved stability, such as liquid-stable or lyophilized mRNA vaccines, will be critical for ensuring reliable distribution in regions with limited cold-chain infrastructure.

Another challenge concerns repeat dosing. For indications such as cancer immunotherapy or chronic infectious disease, multiple administrations are likely to be necessary. However, repeated exposure to PEGylated lipids can trigger anti-PEG antibody responses, leading to accelerated blood clearance and reduced efficacy. This phenomenon also raises concerns regarding hypersensitivity and safety. Moving forward, systematic evaluation of PEG-related properties—including lipid identity, chain length, and exchange rate—should be incorporated into formal CQA frameworks. At the same time, alternative strategies such as cleavable PEG linkers, PEG replacements (e.g., polysarcosine or zwitterionic polymers), and optimized boosting regimens warrant further investigation to mitigate these risks.

Addressing these future challenges will require coordinated efforts in process engineering, formulation design, and immunological standardization. Progress in these areas will not only enable global access but also support the safe extension of mRNA vaccines into diverse therapeutic indications.

## Data Availability

No datasets were generated or analyzed during the current study.
